# Revision of the stiletto fly genera *Acupalpa* Kröber and *Pipinnipons* Winterton (Diptera, Therevidae, Agapophytinae) using cybertaxonomic methods, with a key to Australasian genera

**DOI:** 10.3897/zookeys.95.1461

**Published:** 2011-05-04

**Authors:** Shaun L. Winterton

**Affiliations:** California State Collection of Arthropods, California Department of Food & Agriculture, Sacramento, CA, USA

**Keywords:** cybertaxonomy, LSID, character matrix, natural language description

## Abstract

Australian stiletto flies of the sister-genera *Acupalpa* Kröber, 1912 and *Pipinnipons* Winterton, 2001 (Diptera: Therevidae: Agapophytinae) are revised. Twelve new species of *Acupalpa* are described, while *Acupalpa imitans* (White, 1915), **comb. n.** is transferred from *Pipinnipons* and *Acupalpa albimanis* (Kröber, 1914), **comb. n.** is transferred from *Ectinorhynchus* Macquart as a senior synonym of *Acupalpa pollinosa* Mann. The total number of species of *Acupalpa* is therefore increased to 19: *Acupalpa albimanis* (Kröber), **comb. n.**, *Acupalpa albitarsa* Mann, *Acupalpa boharti* **sp. n.**, *Acupalpa divisa* (Walker), *Acupalpa dolichorhyncha* **sp. n.**, *Acupalpa glossa* **sp. n.**, *Acupalpa imitans* (White), **comb. n.**, *Acupalpa irwini* Winterton, *Acupalpa melanophaeos* **sp. n.,** *Acupalpa miaboolya* **sp. n.**, *Acupalpa minuta* **sp. n.**, *Acupalpa minutoides* **sp. n.**, *Acupalpa notomelas* **sp. n.**, *Acupalpa novayamarna* **sp. n.**, *Acupalpa rostrata* Kröber, *Acupalpa semirufa* Mann, *Acupalpa westralica* **sp. n.**, *Acupalpa yalgoo* **sp. n.** and *Acupalpa yanchep* **sp. n.** Three new species of *Pipinnipons* are described, increasing the total number of species to five: *Pipinnipons chauncyvallis* **sp. n.**, *Pipinnipons fascipennis* (Kröber), *Pipinnipons kampmeierae* **sp. n.**, *Pipinnipons kroeberi* Winterton, and *P. sphecoda* **sp. n.** *Pipinnipons* and *Acupalpa* are rediagnosed in light of the new species presented herein and revised keys to species are included. A dichotomous key to genera of Australasian Therevidae is included. As an empirical example of cybertaxonomy, taxonomic descriptions were composed using a character matrix developed in Lucid Builder (in Structured Descriptive Data (SDD) format) to generate natural language descriptions supplemented by online specimen and image databases. Web resources are provided throughout the document including: a) links to high resolution colour images of all species on Morphbank, b) registration of authors, publications, taxon names and other nomenclatural acts in Zoobank, with assignment of Life Science Identifiers (LSIDs) for each, c) links to Genbank accession records for DNA sequences, and d) assignment of LSIDs to specimen records with links to respective records in an online Therevidae specimen database.

## Introduction

The stiletto fly subfamily Agapophytinae is comprised of 23 described genera restricted in the Australasia region, yet with three additional described genera endemic to Chile and Argentina (Winterton 2006). *Acupalpa* Kröber, 1912 and *Pipinnipons* Winterton, 2001 are agapophytine sister genera found exclusively in Australia. Along with *Agapophytus* Guérin, 1831, these genera form a relatively derived clade characterized by an antenna with an elongate cylindrical scape and flagellum (Winterton 2000; Winterton et al. 2001). *Agapophytus* is differentiated from these genera by an elongate scape typically longer than, or equal to, the length of the flagellum, while in *Acupalpa* and *Pipinnipons* the scape is shorter than the flagellum. *Acupalpa* contains seven previously described species: *Acupalpa albimanis* (Kröber, 1914), comb. n. (=*Acupalpa pollinosa* Mann, 1929, syn. n.), *Acupalpa albitarsa* Mann, 1929, *Acupalpa divisa* (Walker, 1850), *Acupalpa imitans* (White, 1915), comb. n., *Acupalpa irwini* Winterton, 2000, *Acupalpa rostrata* Kröber, 1912, and *Acupalpa semirufa* Mann, 1929. *Pipinnipons* includes two previously described species: *Pipinnipons fascipennis* (Kröber, 1928) and *Pipinnipons kroeberi* Winterton, 2001.

Twelve new species of *Acupalpa* are described herein: *Acupalpa boharti* sp. n., *Acupalpa dolichorhyncha* sp. n., *Acupalpa glossa* sp. n., *Acupalpa melanophaeos* sp. n., *Acupalpa miaboolya* sp. n., *Acupalpa minuta* sp. n., *Acupalpa minutoides* sp. n., *Acupalpa notomelas* sp. n., *Acupalpa novayamarna* sp. n., *Acupalpa westralica* sp. n., *Acupalpa yalgoo* sp. n., and *Acupalpa yanchep* sp. n. Many of these species are from Western Australia, indicating a rich diversity of this genus in the western region of the continent. Addition of these new species significantly broadens the concept of *Acupalpa* beyond the characters defining the genus in previous treatments (i.e. Mann 1929; Winterton 2000; Winterton et al. 2001), therefore the genus and constituent species is rediagnosed herein and a revised key to species presented. *Acupalpa imitans* (White, 1915), comb. n. is transferred from *Pipinnipons* based on the discovery of new material matching the original description, while *Acupalpa albimanis* (Kröber, 1914), comb. n. is transferred from *Ectinorhynchus* Macquart, 1850 as the latter is a senior subjective synonym of *Acupalpa pollinosa*. Three new species of *Pipinnipons* are described, increasing the total number of species to five: *Pipinnipons chauncyvallis* sp. n., *Pipinnipons fascipennis*, *Pipinnipons kampmeierae* sp. n., *Pipinnipons kroeberi*, and *Pipinnipons sphecoda* sp. n. A key to Australasian stiletto fly genera is also included.

The modern taxonomic enterprise represents a gradual paradigm shift away from tedious traditional methods toward rapid, semi-automated ones (i.e. recently termed cybertaxonomy), with increased efficiency in data handling through the use of online databases for information such as label metadata, specimen images, name registration, semantic mark-up and natural language descriptions from character matrices (Winterton 2009). The concept itself is not new, with single zoological registries (e.g. Brown 1961) and rapid descriptive processes (e.g. Erwin and Johnson 2000) espoused previously, but the actual empirical use of informatics tools to enhance the taxonomic descriptive process (i.e. online databases) is only now becoming a reality. Several authors have embraced modern cybertaxonomic methods through the incorporation of such digital, web-based, resources in taxonomic descriptions (e.g. Pyle et al. 2008; Johnson et al. 2008; Deans and Kawada 2008; Miller et al. 2009; Winterton 2009; Penev et al. 2010; Blagoderov et al. 2010). The methods used by these authors, and this paper, are empirical examples of how digital tools can significantly speed the process of documenting biodiversity through rapid generation of natural language descriptions derived from matrix based character data in a standardized format suitable for multiple use (e.g. distributed morphological ontologies).

## Material and methods

Adult morphological terminology follows McAlpine (1981) as modified by Winterton et al. (1999a) and Winterton (2006). Hauser and Irwin (2003) provide a convincing argument for the use of the term pubescence (*sensu* Nichols 1989) instead of pruinescence (*sensu* Winterton et al. 1999a) to describe the various types of microtrichia covering the adult body and is used here throughout the text. The term velutum (Winterton et al. 1999a) is retained to describe a particular type of very dense, unidirectional and reflective (i.e. velvet-like) microtrichia typically found on the male abdomen (silver coloured) or on the femora of agapophytine therevids (variously coloured but usually dark). Genitalia were macerated in 10% KOH to remove soft tissue, then rinsed in distilled water and dilute glacial acetic acid, and dissected in 80% ethanol. Genitalia preparations were placed in glycerine in a genitalia vial mounted on the pin beneath the specimen.

Types are deposited in the following institutions and collections: Queensland Museum (Brisbane) (QM), Australian Museum (Sydney) (AMS), Australian National Insect Collection (Canberra) (ANIC), Senckenberg Deutsches Entomologisches Institut, Müncheberg, Germany (DEI), University of Queensland Insect Collection (Brisbane) (UQIC), University of California, Davis, Bohart Museum (UCDC), Western Australian Museum (Perth) (WAM), Michael E. Irwin private collection [to be ultimately housed in the California Academy of Sciences] (MEIC/CAS), Greg Daniels private collection [to be ultimately housed in the Australian Museum] (GDCB/AMS), Naturhistorisches Museum Wien, (NMW), Harvard University Museum of Comparative Zoology (MCZ), Oxford University Museum of Natural History (OUMNH), University of California, Riverside (UCR), Universität von Hamburg Zoologisches Institut und Zoologisches Museum (ZMUH). All types have been examined. Numbers quoted with individual specimens as MEI000000 are unique identifiers in the therevid database MANDALA and are attached to each specimen as a yellow or white label (Kampmeier and Irwin 2009). Links are provided in this document to Life Science Identifiers (LSIDs) to specimen records with links to respective records in an online MANADALA Therevidae specimen database and Discover Life (http://www.discoverlife.org). Note that some web browsers are not able to read and format RSS (Really Simple Syndication) feeds and/or XML without additional software extensions or plug-ins. Details of current issues with select web browsers and LSID resolvers can be found on the Biodiversity Information Standards (TDWG) LSID resolver website (http://lsid.tdwg.org/). Material examined lists were exported from MANDALA. Descriptions were constructed using Lucid Builder 3.5, using a matrix database of character states, which were then exported using a natural language function into XML and a text document. Links are provided to Genbank accession records for DNA sequences where available. Specimen images were taken using a digital camera with a series of images montaged using Helicon Focus (©HeliconSoft). Descriptions are aided by the provision of embedded URL links in the document to high-resolution digital images of all species in Morphbank. All nomenclatural acts are registered in Zoobank as per the recent proposed amendment to the International Code of Zoological Nomenclature for a universal register for animal names (Polaszek et al.2005a,b; Pyle et al. 2008; ICZN 2008).

## Key to genera of Australasian Therevidae

The following key to genera supersedes those by Winterton et al. (1999b) and Winterton et al. (2001) and includes all genera found throughout the region east of Wallace’s Line, incorporating landmasses such as Australia, New Zealand, New Caledonia, Papua New Guinea, and eastern Indonesia. The enigmatic *Taenogera* genus group (sensu Winterton et al. 1999b) is herein included within an expanded concept of Agapophytinae (Winterton 2006). The subfamilies Xestomyzinae and Phycinae are absent from the Australasian region.

**Table:** 

1	Femora with multiple vestiture types, often with appressed, scale-like setae; strong macrosetae usually present on femora; two spermathecae in female, ventral apodeme of parameral sheath not forked, usually narrow, dorsal apodeme of parameral sheath well developed, usually broad and hood-like	subfamily **THEREVINAE – 2**
–	Femora with only a single type of setae, often short and dark setae admixed with longer pale setae, never appressed or scale-like, macrosetae sometimes present; three spermathecae (rarely reduced to one or two); ventral apodeme of parameral sheath forked or anteriorly emarginate, never as single narrow apodeme; dorsal apodeme of parameral sheath usually forked or greatly reduced, rarely broad or hood-like	subfamily **AGAPOPHYTINAE – 4**
2	Male frons narrow so that eyes almost contiguous medially; usually a single row of postocular setae dorsomedially in male; mid coxal pile present; wing cell m3 open or closed; femora with distinct appressed pile (Indonesia, Papua New Guinea) (relatively slender flies)	*Irwiniella* Lyneborg
–	Male frons usually wider than ocellar tubercle so that eyes widely separated; usually many rows of postocular setae dorsally in both sexes; mid coxal pile absent (rarely present in *Anabarhynchus*); wing cell m3 open; femora with multiple vestiture types, sometimes without distinct appressed pile (robust flies)	3
3	Size variable, but never uniformly black; (Australia, Papua New Guinea, New Caledonia, Fiji, New Zealand)	*Anabarhynchus* Macquart
–	Large, black, robust flies; female sternite 8 with posterolateral slits (New Zealand)	*Megathereva* Lyneborg
4	Elongate strip of velutum (velvet pubescence) on ventromedial surface of hind femur present; patch of velutum on ventral surface of male gonocoxite often present (rarely reduced or absent); wing cell m3 open or closed (Australia, Papua New Guinea, Indonesia)	5
–	Femora without of velutum patches; gonocoxites without velutum patch ventrally (rarely present); wing cell m3 open (Australia, New Zealand, Papua New Guinea, Indonesia, New Caledonia)	18
5	Wing cell m3 open	6
–	Wing cell m3 closed	9
6	Fore femur without velutum patch on ventral surface; hind femur with one (rarely more) subapical anteroventral seta; antennae usually longer than head, flagellum cylindrical; occiput often overlain with silver, gold and matte black pubescence (Australia)	*Evansomyia* Mann
–	Fore femur with velutum patch on ventral surface; hind femur without subapical setae; antennae usually shorter than head, flagellum conical; occiput not overlain with silver, gold and matte black pubescence	7
7	Short, relatively small flies; male genitalia with ventral lobe of male gonocoxite not broad or enlarged; medial atrium (Winterton et al. 2001: fig 22) usually present (Australia)	*Parapsilocephala* Krober (part)
–	Elongate, small to relatively large flies; ventral lobe of gonocoxite broad, enlarged; medial atrium absent	8
8	Eyes relatively small; occiput concave, postocular ridges angled, not in same plane; relatively few postocular setae, arranged in a poorly defined single row; setae absent on posterior surface of mid coxa; postspiracular setae absent; mid femur without elongate velutum patch; distiphallus spinose apically (Australia)	*Belonalys* Krober
–	Eyes regular size; occiput concave or convex, postocular ridges straight or almost in same plane, usually with multiple rows of postocular setae dorsally, some males with only a single row; setae sometimes present on prosternum, and on posterior surface of mid coxa; postspiracular setae sometimes present; additional elongate velutum patch on posteroventral surface of mid femur often present; distiphallus without spines (Australia)	*Laxotela* Winterton & Irwin
9	Flagellum elongate, narrow cylindrical in cross-section; scape also narrow elongate, usually more than 3? length of pedicel; antennae typically longer than head (rarely equal in length); antennae appear positioned on middle or upper region of head, rarely on lower frons; antennae usually not projecting anteroventrally to body axis	10
–	Flagellum conical, turbinate or oval shaped, usually flattened laterally; scape length variable but never elongate and narrow cylindrical, usually less than 3? pedicel length, sometimes bulbous; antennae shorter than head, although sometimes close to equal head length, antennae positioned very low on head and projecting anteroventrally to body axis	12
10	Flagellum shorter than or equal to scape length; scape usually longer than head (Australia, Papua New Guinea)	*Agapophytus* Guerin
–	Flagellum longer than scape length; scape never longer than head	11
11	Palpi spatulate; face narrow, not expansive or protruding (Australia)	*Pipinnipons* Winterton
–	Palpi narrow to acuminate, not broadened apically; face expansive and often protruding anteriorly (Australia)	*Acupalpa* Krober
12	Black individuals with silver-white velutum stripe along lower half of thorax and abdomen; male genitalia with articulated gonocoxal process absent or greatly reduced; ventral lobe greatly enlarged into blade-like structure (Australia)	*Vomerina* Winterton
–	Body colour and markings otherwise (silver velutum stripe on pleuron present in some genera); male genitalia with well-developed articulated gonocoxal process; ventral lobe not large and blade-like	13
13	Wing typically strongly banded; abdomen slender and narrow basally, diameter of thorax distinctly greater than base of abdomen; hind femur longer than fore and mid femora; male genitalia with medial atrium present or absent	14
–	Wing hyaline or variably infuscate, but not banded; abdomen thicker basally, diameter similar to thorax, slightly tapered posteriorly but not slender; femora approximately equal length male genitalia with medial atrium present	16
14	Male epandrium arched to partially conceal gonocoxites; medial atrium present between gonocoxites; hypandrium with patch of strong posteriorly directly setae (Australia)	*Acatopygia* Krober
–	Male epandrium not concealing gonocoxites; gonocoxites meeting medially such that medial atrium is absent; hypandrium without patch of strong setae	15
15	Small species (usually <6.0 mm body length); scutellum often dorsally acuminate; frequently excellent ant mimics; male with single row of postocular setae (Australia, Papua New Guinea, Timor)	*Acraspisa* Krober
–	Relatively larger species (8.0–12.0 mm body length), usually larger than 6.0mm total body length; scutellum rounded, never dorsally acuminate; male with multiple rows of postocular setae (Australia)	*Acraspisoides* Hill & Winterton
16	Ventral lobe very long and narrow, length equal to gonostylus; plate or cup-like velutum patch on gonocoxites; frons flattened or rounded, without callus above antenna (Australia)	*Patanothrix* Winterton
–	Ventral lobe shorter than gonostylus; velutum barely evident on gonocoxite, found mainly on atrium membrane; frons typically with glossy callus above antenna	17
17	Three spermathecae present; transverse velutum plaques absent on male abdomen; gonocoxal apodemes and distiphallus short; ejaculatory apodemes strongly sclerotized but not enlarged (Australia)	*Parapsilocephala* Krober (part)
–	One spermatheca present; transverse velutum plaques often present on male abdomen; gonocoxal apodemes and distiphallus often greatly elongate; ejaculatory apodemes greatly enlarged (Australia)	*Bonjeania* Irwin and Lyneborg
18	Hind femur without subapical setae	19
–	Hind femur with one (rarely more) subapical anteroventral seta	20
19	Male gonocoxite with processes absent; male usually with a single row of postocular setae dorsally; medium sized flies; colouration and markings variable, scutum often yellow or tan ground colour with dark brown tessellate or spotted pattern (Australia)	*Neodialineura* Mann
–	Male gonocoxite with gonocoxal process present; male usually with multiple poorly defined rows of postocular setae dorsally; relatively small flies; grey and black colouration and markings (Australia)	*Manestella* Metz
20	Body usually large to medium sized, robust, glossy dark metallic blue or orange; abdomen abruptly tapered; small patch of postspiracular setae present on thorax; wing extensively black (sometimes hyaline basally) or orange infuscate	21
–	Body size variable, usually relatively slender, never glossy metallic blue or orange; abdomen elongate, evenly tapered; thoracic postspiracular setae absent; wing infuscation variable, usually banded or hyaline, never uniform orange or black	22
21	Scape short, setae on antennae and head relatively short; two pairs of scutellar setae; wing with uniform orange infuscation (Australia)	*Eupsilocephala* Krober
–	Scape elongate with numerous enlarged setae; single pair of scutellar setae; wing either with uniform black infuscation or hyaline basally (Australia)	*Johnmannia* Irwin and Lyneborg
22	Male and female occiput convex, variously overlain with bronze, matte black, silver and gold pubescence; multiple rows of postocular setae in male; abdomen of equal diameter along length; distiphallus broad, cylindrical; medium to large individuals (Australia)	*Taenogera* Krober
–	Male occiput typically flat to concave, not distinctly convex, rarely overlain with bronze, matte black, silver and gold pubescence; usually single row of postocular setae in male; abdomen tapered; distiphallus usually narrow; size variable	23
23	Antennae shorter than or equal to head length; scape usually < 2? pedicel length, usually with only small setae on scape and frons	24
–	Antennae longer than head; scape > 3? pedicel length, often with strong setae on scape and frons	25
24	Occiput with multiple rows of postocular setae in both sexes; male with articulated gonocoxal process greatly reduced or absent; gonocoxite sometimes with large horn-like posterior process (Australia)	*Actenomeros* Winterton & Irwin
–	Occiput with single row of postocular setae in male; male with articulated gonocoxal process well developed; gonocoxite without horn-like posterior process (Australia)	*Nanexila* Winterton & Irwin
25	Occiput overlain with silver and matte black pubescence; male with single row of postocular setae; typically larger species with banded wings	26
–	Occiput overlain with grey pubescence; male often with multiple rows of postocular setae; smaller species with hyaline or slightly suffused wings (Australia)	*Taenogerella* Winterton & Irwin
26	Male with medial atrium between gonocoxites, articulated gonocoxal process greatly reduced; velutum patch present ventrally on gonocoxites; colouration and markings often sexually dimorphic (Australia, New Zealand)	*Ectinorhynchus* Macquart
–	Male without medial atrium between gonocoxites, articulated gonocoxal process well developed; velutum absent on gonocoxites; colouration and markings not sexually dimorphic (Australia, Papua New Guinea)	*Squamopygia* Krober

## Taxonomy

### Acupalpa

Kröber

urn:lsid:zoobank.org:act:68450BF4-0179-4194-BE8D-422966FC95C7

http://species-id.net/wiki/Acupalpa

Acupalpa Kröber 1912: 152; Kröber 1913: 18; Mann 1929: 23; Hardy 1939: 47 [as *Acutipalpa*]; Irwin and Lyneborg 1989: 354 [catalogue]; Winterton 2000: 227 [revision]; Winterton et al. 2001: 197. Type species: *Acupalpa rostrata*Kröber 1912: 152.

#### Diagnosis.

Antennal scape shorter than or equal to flagellum; antenna elongate, cylindrical, total length slightly longer than or equal to head length; upper part of frons flat or slightly concave above antenna; face either protruding anteriorly below antennal base, or broadly rounded, expansive, short dark setae often present; parafacial setae absent; palpus apically narrow or acute, not spatulate; mouthparts length variable, frequently elongate and forward projecting (Fig. 3H); male postocular ridge with single row of macrosetae immediately laterad of ocellar tubercle, female with more than one row; wing infuscate, usually strongly banded; setae absent on wing vein R1; cell m3 closed; velutum patches on fore and hind femora; femora without macrosetae; single type of setal pile on femora, setae not appressed; prosternal furrow without setae; post spiracular pile absent; pleuron orange to black, overlain with sparse silver pubescence; mid coxa without setae on posterior surface; gonocoxites with velutum patch on ventral surface (Fig. 3B); articulated gonocoxal process present; hypandrium present; ventral apodeme of parameral sheath forked; dorsal apodeme of parameral sheath ‘T’-shaped (Fig. 3F); three spermathecae in female; spermathecal sac present, sac simple or with smaller additional lobes basally, often with outer elongate lobes; spermathecal ducts joining common duct before bursa (Fig. 3G), female with A1 and A2 acanthophorite spines well developed; female sternite 8 emarginate along posterior margin.

**Figure 1.**
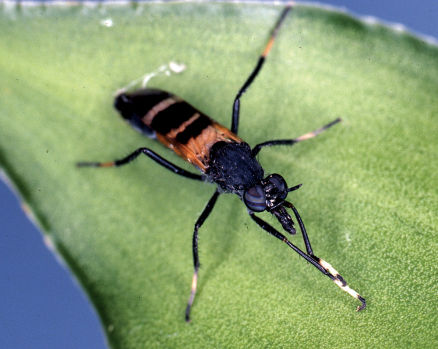
*Acupalpa divisa* (Walker), female, Brisbane, Queensland. Body length= 7.0 mm. (Photo: Anthony O’Toole, University of Queensland).

**Figure 2.**
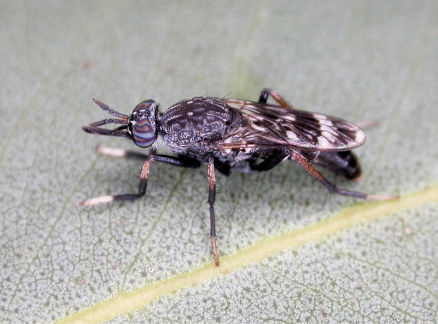
*Acupalpa yanchep* sp. n., female, Yanchep, Western Australia. Body length= 9.0 mm. (Photo: S.L. Winterton).

**Figure 3.**
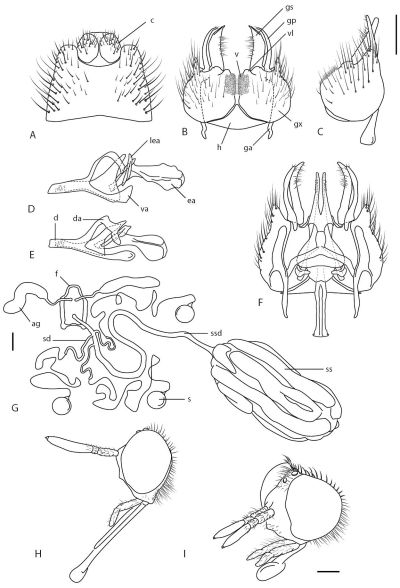
*Acupalpa* spp.: **A** *Acupalpa notomelas* sp. n., epandrium dorsal **B** gonocoxites, ventral **C** *Acupalpa imitans* (White), comb. n., gonocoxite, lateral **D** same, aedeagus, lateral **E** *Acupalpa rostrata* Kröber aedeagus, lateral **F** *Acupalpa imitans*, gonocoxites, epandrium removed and aedeagus *in situ*, dorsal **G** *Acupalpa rostrata*, female spermathecal sac complex, dorsal **H** *Acupalpa melanophaeos* sp. n., female head, lateral **I** *Acupalpa rostrata*, male head, anterolateral. Abbreviations: **ag** accessory gland **c** cercus **d** distiphallus **da** dorsal apodeme of parameral sheath **ea** ejaculatory apodeme **f** furca **ga** gonocoxal apodeme **gp** (articulated) gonocoxal process **gs** gonostylus **gx** gonocoxite **h** hypandriumgp gonocoxal process (articulated) **lea** lateral ejaculatory apodeme **s** spermatheca **ss** spermathecal sac **ssd** spermathecal sac duct **va** ventral apodeme of parameral sheath **v** velutum patch **vl** ventral lobe. Scale lines = 0.2 mm.

#### Comments.

*Acupalpa* is a genus with some distinctive wasp mimicking species (Figs 1–2), often strikingly coloured with black and orange. The male terminalia are relatively conserved throughout both *Acupalpa* and *Pipinnipons*, and species identification is more easily and reliably accomplished using external characters of either sex. Closely related to *Pipinnipons* and *Agapophytus*, *Acupalpa* can be distinguished by the elongate, cylindrical antennae, scape not longer than flagellum, face usually expansive and protruding, and palpi that are acuminate or narrowly cylindrical. The latter two characters specifically differentiate *Acupalpa* from *Pipinnipons*, as the face is always narrow and the palpi spatulate in *Pipinnipons*. *Agapophytus* is separated from *Pipinnipons* and *Acupalpa* by the length of the scape ranging from relatively equal length, to significantly longer than the flagellum.

#### Included species.

*Acupalpa albimanis* (Kröber), comb. n., *Acupalpa albitarsa* Mann, *Acupalpa boharti* sp. n., *Acupalpa divisa* (Walker), *Acupalpa dolichorhyncha* sp. n., *Acupalpa glossa* sp. n., *Acupalpa imitans* (White), comb. n., *Acupalpa irwini* Winterton, *Acupalpa melanophaeos* sp. n., *Acupalpa miaboolya* sp. n., *Acupalpa minuta* sp. n., *Acupalpa minutoides* sp. n., *Acupalpa notomelas* sp. n., *Acupalpa novayamarna* sp. n., *Acupalpa rostrata* Kröber, *Acupalpa semirufa* Mann, *Acupalpa westralica* sp. n., *Acupalpa yalgoo* sp. n., *Acupalpa yanchep* sp. n.

#### Key to *Acupalpa* species

**Table:** 

1	Abdomen ground colour completely brown to black (terminal segments sometimes orange in female) (e.g. Figs 5^10^,16)	2
–	Abdomen with any number of segments 1–3 completely or partially orange or yellow (e.g. Figs 7–9,11–12)	11
2	Fore and mid coxae brown to black, overlain with silver-grey pubescence (e.g. Figs 5–6,13,18)	4
–	Fore and mid coxae orange to pale yellow (Figs 16^20^)	3
3	Lower pleuron orange; fore femur brown; base of mid and hind femora orange; foreleg second tarsomere pale (Fig. 16)	*Acupalpa notomelas* sp. n.
–	Pleuron uniformly brown to black; fore and mid femora brown to black dorsally, pale ventrally; hind femur brown to black; foreleg second tarsomere brown to black (female only) (Fig. 20)	*Acupalpa westralica* sp. n.
4	Scape and pedicel yellow; fore and mid femora yellow (Fig. 18)	*Acupalpa rostrata* (Krober)
–	Scape and pedicel brown to black (rarely pale basally); fore and mid femora brown to black	5
5	Very small sized species (< 3.5 mm total body length); pleuron with longitudinal stripe of silver velutum; flagellum grossly enlarged (> 3 time combined scape and pedicel length) (Fig. 14); two notopleural macrosetae	6
–	Size variable, but usually larger than 5.0 mm; pleuron colour and vestiture variable but never with longitudinal silver velutum stripe; flagellum length sub-equal to 0.5 times combined scape and pedicel length; always more than two notopleural macrosetae	7
6	Coxal macrosetae pale; frons width slightly narrower than ocellar tubercle width; wing vein M1 and M2 originating separately from discal cell; fore tibia uniformly brown to black (Fig. 14) (male only)	*Acupalpa minuta* sp. n.
–	Coxal macrosetae black; male frons slightly wider than ocellar tubercle width; wing vein M1 and M2 fused basally and originating as a petiolate stem from discal cell; fore tibia yellowish basally (Fig. 15) (male only)	*Acupalpa minutoides* sp. n.
7	Fore tibia pale yellow to white, rarely brown distally; male abdomen without silver velutum; small species (Figs 6^13^)	8
–	Fore tibia dark, rarely pale basally; male abdomen with silver velutum; larger species (Figs 5^22^)	9
8	Scutum overlain with mostly uniform brown-bronze pubescence; fore tibia uniform white-cream (Fig. 6)	*Acupalpa boharti* sp. n.
–	Scutum overlain with grey and brown striped pubescence; fore tibia white-cream, but darker basally (Fig. 13)	*Acupalpa miaboolya* sp. n.
9	Basitarsi white to cream, fore-basitarsus darker basally, all tarsomeres 3–5 dark (Fig. 4)	*Acupalpa albimanis* (Krober), comb. n.
–	Basitarsi brown to black, tarsomeres 2–5 white	10
10	Male frons at narrowest point slightly narrower than width of ocellar tubercle (western Australia) (Figs 2^22^)	*Acupalpa yanchep* sp. n.
–	Male frons approximately equal width to ocellar tubercle at narrowest point (eastern Australia) (Fig. 5)	*Acupalpa albitarsa* Mann
11	Femora pale yellow to orange (Figs 9–10,12,17,19)	12
–	Femora brown to black (Figs 7–8,11,21)	16
12	Coxae pale orange; pleuron dark with orange suffusion; male abdomen without silver velutum (Figs 12^17^)	13
–	Coxae dark (rarely dark orange), overlain with silver-grey pubescence; pleuron completely dark, overlain with silver pubescence; male abdomen with or without silver velutum dorsally (Figs 9–10,19)	14
13	Pleuron mostly dark; scutal setae minute; genal setae dark; male frons width equal to width of anterior ocellus (Fig. 12)	*Acupalpa melanophaeos* sp. n.
–	Pleuron mostly orange; scutal setae medium length; genal setae pale; male frons width equal to width of ocellar tubercle (Fig. 17)	*Acupalpa novayamarna* sp. n.
14	Tibia and tarsi mostly darkened apically; frons profile concave; male abdomen with dense silver velutum dorsally (Fig. 19)	*Acupalpa semirufa* Mann
–	Tibia and tarsi without distinctive darkening apically; frons profile rounded; male abdomen without dense silver velutum dorsally	15
15	Abdominal segments 2–3 bright orange, with orange suffusion of segments 1 and 4; haltere knob brown (Fig. 9)	*Acupalpa glossa* sp. n.
–	Abdominal segments 2–3 slight orange-tan laterally; haltere knob white (Fig. 10)	*Acupalpa imitans* (White), comb. n.
16	Tibia brown to black (Figs 7–8)	17
–	Tibia yellow to cream basally, dark apically (Figs 11^21^)	18
17	Mouthparts greatly elongate, much longer than head length; foreleg basitarsus entirely white to cream (Fig. 8)	*Acupalpa dolichorhyncha* sp. n.
–	Mouthparts approximately equal to head length; foreleg basitarsus darker basally, pale distally (Fig. 7)	*Acupalpa divisa* (Walker)
18	Antennal scape and face with short, dark setae; face flat, not protruding anteriorly (male only) (Fig. 21)	*Acupalpa yalgoo* sp. n.
–	Antennal scape and face with pale setae; face protruding anteriorly (Fig. 11)	*Acupalpa irwini* Winterton

### Acupalpa albimanis

(Kröber) comb. n.

urn:lsid:zoobank.org:act:911E11F8-66AF-41CF-9B32-8C4B4D744C6A

http://species-id.net/wiki/Acupalpa_albimanis

Fig. 4

Ectinorhynchus albimanus Kröber 1914: 31. *-*Irwin and Lyneborg 1989: 356; *nec*. Mann 1928: 156; 1933: 334.
Acupalpa pollinosa Mann 1929: 25; Hardy 1939: 47 [as *Acutipalpa polinosa*]; Irwin and Lyneborg 1989: 354 [catalogue]; Winterton 2000: 235; Winterton et al. 2001: 210. syn. n.

#### Type material.

*Ectinorhynchus albimanus* Kröber, 1914 *-* **Holotype** female ‘N. Holl. [Neu Holland] 878 IV/ TYPE (ANIC29_003432) (NMW).

*Acupalpa pollinosa* Mann, 1929 *-* **Holotype** male, AUSTRALIA: **Queensland:** Brisbane, 18.ix.1914, H. Hacker (MEI029468) **[**D3283] (QM). **Paratypes:** AUSTRALIA: **Queensland:** 2 males, Brisbane, 24.ix.1914, 24.ix.1923, H. Hacker (MEI108792, 108793) (QM).

#### Diagnosis.

Frons profile concave above antenna; antenna black; wing dark banded; legs black with basitarsus and second tarsomere white; abdomen black, overlain with silver velutum in male.

#### Redescription.

Body length= 6.9–9.3 mm. *Head*. Frons wider than ocellar tubercle, profile transversely concave above antennae, pubescence as two silver patches along eye margin, vestiture as minute setae; frons surface texture as irregular longitudinal striations; face projecting anteriorly, vestiture with dark to pale setae; gena with pale setae; parafacial glabrous; mouthparts relatively short (approximately equal to head length); palpus brown-black, acuminate; occiput glabrous, glossy black; antennal base raised; antennal length approximately equal to head; scape brown to black, length much shorter than flagellum, with sparse black setae ventrally; flagellum black, base of flagellum with short, dark setae. *Thorax*. Scutum uniform grey-black; scutellum overlain with dense, matt-black pubescence; pleuron black, overlain with sparse silver-grey pubescence; wing markings dark banded infuscate; haltere knob white, stem dark brown; coxae and femora brown to black; tibia dark; tarsi dark, basal ¾ of fore-basitarsus and entire second tarsomere cream to white. Scutal chaetotaxy (macrosetae pairs): *np* (notopleural), 4; *sa* (supra-alar), 1; *pa* (post-alar), 1; *dc* (dorsocentral), 2–3; *sc* (scutellar), 1. *Abdomen*. Black, covered with silver velutum dorsally on tergites (male only); terminalia pale.

**Figure 4.**
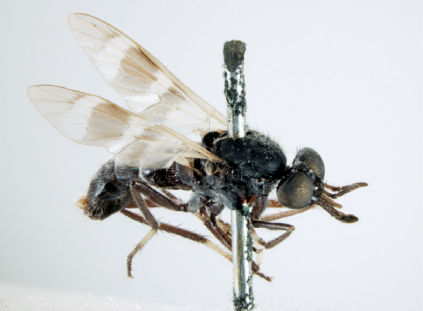
*Acupalpa albimanis* (Kröber), comb. n., (Holotype of *Acupalpa pollinosa*), male, anterolateral view [Morphbank: 576222]. Body length = 6.5 mm.

#### Comments.

*Ectinorhynchus albimanis* is herein transferred to *Acupalpa* with *Acupalpa pollinosa* becoming a junior synonym of *Acupalpa albimanis* comb. n. Mann (1928) redescribed *Ectinorhynchus albimanis* based on a series of specimens, but clearly did not examine the type, as his redescription does not match that in Kröber (1914) nor reflect characteristics of the type. *Acupalpa albimanis* is morphological similar to *Acupalpa albitarsa* and *Acupalpa yanchep* sp. n. The colouration of abdomen and tarsi is diagnostic for this species.

### Acupalpa albitarsa

Mann

urn:lsid:zoobank.org:act:44480644-4D44-460F-9092-7E610A4767F0

Genbank Accession: AF150967

http://species-id.net/wiki/Acupalpa_albitarsa

Fig. 5

Acupalpa albitarsa Mann 1929: 24; Irwin and Lyneborg 1989: 354 [catalogue]; Winterton 2000: 230; Winterton et al. 2001: 210.

#### Type material.

**Holotype** male, AUSTRALIA: **Queensland:** Brisbane, 24.ix.1914, H. Hacker [-27.465, 153.017] (MEI029448) **[**D3282**]** (QM). **Paratypes:** AUSTRALIA: **Queensland:** 4 males, same data as holotype, (MEI108766, 108768, 108770, 108771) (QM).

#### Diagnosis.

Frons profile rounded above antenna; antenna black, scape sometimes brown; wing irregularly banded; pleuron black; tarsi white with brown to black basitarsus; abdomen with sparse silver velutum on anterior segments (denser in male).

#### Redescription.

Body length= 7.4–9.7 mm. *Head*. Frons wider than ocellar tubercle; profile rounded above antenna, pubescence sparse silver-grey; frontal vestiture as minute setae, texture verrucous; face shape broadly rounded, vestiture with dark or pale setae; gena with pale setae; parafacial glabrous; mouthparts elongate and projecting anteriorly, or relatively short; palpus brown-black, acuminate; occiput glabrous, glossy black; antennal base raised; antenna longer than head; scape brown or black, length approximately equal to flagellum, with sparse black setae; flagellum black, base of flagellum with short, dark setae. *Thorax*. Scutum light grey to black, setal bases glossy black; scutellum overlain with dense, matt-black pubescence; pleuron black, overlain with sparse, silver-grey pubescence; wing markings irregularly banded; haltere knob white; coxae black; femora brown to black; tibia dark, lighter basally; tarsi white with basitarsi dark. Scutal chaetotaxy: *np*, 3–4; *sa*, 1; *pa*, 1; *dc*, 2–3; *sc*, 1. *Abdomen*. Entirely black, segments 5–8 sometimes orange dorsally; silver velutum dorsally on tergites (1–3), bronze medially; terminalia dark (male) or pale (female).

**Figure 5.**
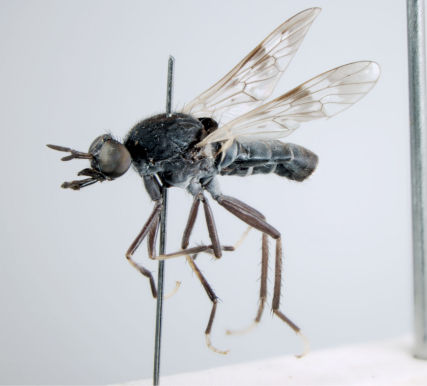
*Acupalpa albitarsa* Mann, male, anterolateral view [576246]. Body length = 7.0 mm.

#### Comments.

The distinctive tarsal colouration of *Acupalpa albitarsa* and the closely related *Acupalpa yanchep* sp. n., separates these species from all other *Acupalpa*. *Acupalpa albitarsa* is an eastern species while *Acupalpa yanchep* sp. n. is western. Females are difficult to distinguish but males differ in the shape of the frons and in general body shape and size. The white patterning of the scutum is less pronounced in this species.

### Acupalpa boharti sp. n.

urn:lsid:zoobank.org:act:8F89524D-85C5-4332-86AC-D278AB724C1D

http://species-id.net/wiki/Acupalpa_boharti

>Fig. 6

#### Type material.

**Holotype** female, AUSTRALIA: **Western Australia:** Norseman, [-32.167°, 121.75°], 24.xi.1979, R. M. Bohart (MEI029500) (UCDC). **Paratypes**. AUSTRALIA: **Western Australia:** male, female, Norseman, [-32.167°, 121.75°], 24.xi.1979, R. M. Bohart (MEI029499, 029501) (UCDC).

#### Diagnosis.

Body size relatively small; frons rounded above antenna; scutum glossy black with bronze pubescence; tibia yellow with dark apices, fore tibia white-cream; abdomen black, without velutum.

#### Description.

Body length= 5.0–6.0 mm. *Head*. Frons wider than ocellar tubercle, profile rounded above antenna, glabrous; frontal vestiture as minute setae, texture smooth; face broadly rounded, glabrous; gena with pale setae; parafacial glabrous; mouthparts elongate, projecting anteriorly, or sometimes relatively short; palpus brown-black, acuminate; occiput glabrous, glossy black; antennal base flat; frons roughly level with eye in profile; antenna longer than head; scape brown or black, length shorter than flagellum, with sparse black setae; flagellum black or brown, base of flagellum with short dark setae. *Thorax*. Scutum glossy black, overlain with sparse bronze pubescence; scutellum overlain with dense, matt-black pubescence; pleuron black, overlain with sparse, silver-grey pubescence; wing markings weakly infuscate with pale band midway, hyaline ocellations basally; haltere knob orange-yellow; coxae black; femora brown to black; tibia yellow, apices dark on mid and hind tibia, fore tibia white-cream; tarsi black; mid and hind basitarsi pale basally. Scutal chaetotaxy: *np*, 4; *sa*, 1; *pa*, 1; *dc*, 4–6; *sc*, 1. *Abdomen*. Black, silver velutum absent, terminalia dark.

**Figure 6.**
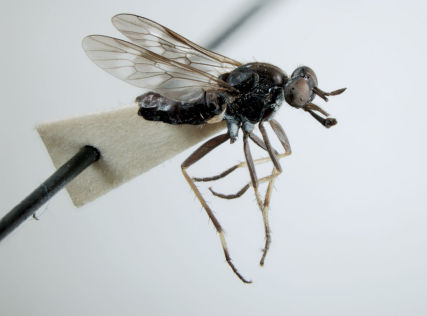
*Acupalpa boharti* sp. n., female, lateral view [576266]. Body length = 5.0 mm.

#### Comments.

*Acupalpa boharti* sp. n. is a small, dark species similar to *Acupalpa miaboolya* sp. n. This species is known only from the type series collected in southwestern Australia. The small body size and leg and body colouration are diagnostic for this species.

#### Etymology.

This species is named in honour of the collector, R. M. Bohart.

### Acupalpa divisa

(Walker)

urn:lsid:zoobank.org:act:318DBAA4-B88A-4276-897B-643F78FA4AA5

Genbank Accession: AF150966

http://species-id.net/wiki/Acupalpa_divisa

Fig. 7

Dimassus divisus Walker 1850: 3.
Ectinorrhynchus divisus (Walker) - Kröber 1913: 19.
Acupalpa semiflava Mann 1929: 28.
Acupalpa divisa (Walker) - Irwin and Lyneborg 1989: 354 [catalogue]; Winterton 2000: 232; Winterton et al. 2001: 210.

#### Type material.

*Dimassus divisus* Walker 1850 *-* **Holotype** female, AUSTRALIA (OUMNH).

*Acupalpa semiflava* Mann 1929 *- ***Holotype** female, AUSTRALIA: **Queensland:** Brisbane, 24.ix.1914, H. Hacker (MEI029471) (QM). **Paratypes:** **Queensland:** 3 females, Brisbane, 24.ix.1912, 14.x.1913, 10.x.1916, H. Hacker (MEI092529, 092532, 092534) (QM).

#### Additional material.

AUSTRALIA: **Queensland:** male, Barakula State Forest, Hellhole Creek, Auburn Rd., 52062, 13.x.2004, Queensland Museum party, open forest, hand collected [-26.33°, 150.7°] (ANIC29_016460) (QM).

#### Diagnosis.

Frons profile concave above antenna; antenna black; pleuron black; wing dark banded; femora and tibia black; abdomen black, segments 1–3 yellow at least laterally; abdominal velutum present in male.

#### Redescription.

Body length= 6.5–8.0 mm. *Head*. Frons wider than ocellar tubercle, profile transversely concave above antennae, pubescence as silver patches along eye margin, frontal vestiture as minute setae, texture as irregular longitudinal striations; face produced anteriorly, vestiture with dark or pale setae; gena with pale setae; parafacial glabrous; mouthparts elongate, projecting anteriorly, or sometimes relatively short; palpus brown-black, acuminate; occiput glabrous, glossy black; antennal base raised, antennal length approximately equal to head; scape black, length approximately equal to flagellum, with sparse black setae; flagellum black, base of flagellum with short dark setae. *Thorax*. Scutum uniform grey-black, sometimes with faint white stripes; scutellum overlain with dense, matt-black pubescence; pleuron black, overlain with sparse silver-grey pubescence; wing markings dark banded infuscate; haltere knob white; coxae black; femora brown to black; tibia black; tarsi black; fore basitarsus white distally, 2nd tarsomere basally, remaining basitarsi yellowish. Scutal chaetotaxy: *np*, 4; *sa*, 1; *pa*, 1; *dc*, 3–4; *sc*, 1. *Abdomen*. Segments 2–3 yellow, remaining segments black (male tergites 1-3 dark medially), silver velutum dorsally on tergites (male) or absent (female); terminalia dark.

**Figure 7.**
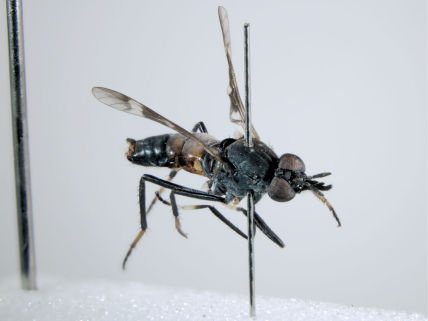
*Acupalpa divisa* (Walker), male, anterolateral view [576245]. Body length = 7.0 mm.

#### Comments.

The male of *Acupalpa divisa* has long been unknown, and herein described for the first time. Hardy (1939) proposed that this species was a synonym of *Acupalpa pollinosa*, but has been subsequently proved incorrect as corresponding sexes of both species are now known.

### Acupalpa dolichorhyncha sp. n.

urn:lsid:zoobank.org:act:56FF95E1-D936-4DBA-8EBF-3A6E79B24116

http://species-id.net/wiki/Acupalpa_dolichorhyncha

Fig. 8

#### Type material.

**Holotype** male, AUSTRALIA: **Western Australia:** 11 km N Cataby, 29.x.1987, M. E. Irwin & E. I. Schlinger, sweeping *Leptospernum* flowers [-30.733°, 115.533°] (MEI029507) (ANIC). **Paratype**. AUSTRALIA: **Western Australia:** female, same data as holotype (MEI029506) (ANIC).

#### Diagnosis.

Mouthparts elongate; frons profile rounded above antenna; antenna black; scutum glossy black; pleuron black; wing dark banded; abdominal segments 1–3 orange, rest black; abdominal velutum absent.

#### Description.

Body length= 7–10 mm. *Head*. Frons wider than ocellar tubercle (male), profile rounded above antenna, pubescence absent, glabrous, frontal vestiture glabrous or as minute setae, surface texture smooth; face shape broadly rounded, expansive, vestiture with dark or pale setae; gena with pale setae; parafacial glabrous; mouthparts elongate, projecting anteriorly; palpus brown-black, narrowly cylindrical; occiput overlain with sparse, silver-grey pubescence; antennal base flat, frons roughly level with eye in profile; antennal length approximately equal to head; scape black with sparse black setae, length shorter than flagellum; flagellum black, base of flagellum with short dark setae. *Thorax*. Scutum glossy black, overlain with faint stripes of grey pubescence; scutellum overlain with dense, matt-black pubescence; pleuron black, overlain with sparse silver-grey pubescence; wing markings faintly banded infuscate; haltere knob white; coxae black; femora brown to black; tibia brown or black; tarsi dark, basitarsi pale, dark distally, fore-basitarsus entirely white. Scutal chaetotaxy: *np*, 4; *sa*, 1; *pa*, 1; *dc*, 2; *sc*, 1. *Abdomen*. Segments 1-3 orange, remaining segments black, silver velutum absent; terminalia dark.

**Figure 8.**
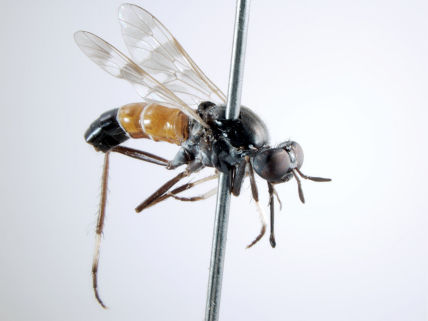
*Acupalpa dolichorhyncha* sp. n., male, anterolateral view [576248]. Body length = 8.0 mm.

#### Comments.

*Acupalpa dolichorhyncha* sp. n. is a distinctive species with elongate mouthparts and orange-banded abdominal segments 1–3. This western species is morphologically similar to *Acupalpa melanophaeos* sp. n., also from Western Australia, and *Acupalpa glossa* sp. n. from Victoria.

#### Etymology.

The specific epithet is derived from Gr. *dolichos*, long; *rhynchus*, snout, referring to the elongate mouthparts.

### Acupalpa glossa sp. n.

urn:lsid:zoobank.org:act:60BC378C-1247-4CB5-A194-755C00BC5567

http://species-id.net/wiki/Acupalpa_glossa

Fig. 9

#### Type material.

**Holotype** male, AUSTRALIA: **Victoria:** 5 km S Rocket Lake, Murray-Sunset N.P., 34.39°S, 141.49°E, 25.xi.1992, swept, McEvey, Moulds, McAlpine (MEI165183) (AMS). **Paratypes**. AUSTRALIA: **Victoria:** male, 2 females, Murray-Sunset N.P., Millewa South Bore track, 20.7 km S Shearers Quarters, 17–23.xi.2002, C. Lambkin, D. Yeates, N. Starick, J. Recsei, 34°45'02"S, 141°03'56"E [Malaise trap] (MEI165184, 165185, 165186) (ANIC).

#### Diagnosis.

Frons profile rounded above antenna; antenna black; scutum glossy black; pleuron orange to brown, darker posteriorly; wing banded infuscate; femora orange; tibia black; abdomen black, without velutum.

#### Description.

Body length= 7.0–9.0 mm. *Head*. Frons wider than ocellar tubercle, profile rounded above antenna, glabrous, sometimes with silver patches of pubescence along eye margin, frontal vestiture as minute setae, surface texture smooth; face broadly rounded, vestiture as dark or pale setae; gena with pale setae; parafacial glabrous; mouthparts elongate, projecting anteriorly; palpus brown-black, narrowly cylindrical; occiput overlain with sparse, silver-grey pubescence; antennal base flat; antennal length approximately equal to head; scape brown, shorter than flagellum, with sparse black setae; flagellum black, base of flagellum with short dark setae. *Thorax*. Scutum black, overlain with grey pubescence; scutellum overlain with dense, matt-black pubescence; pleuron dark, overlain with sparse silver-grey pubescence, denser anteriorly and posteriorly, sparse around midway; wing banded infuscate; haltere knob white; coxae dark, overlain with dense silver pubescence; femora orange; tibia orange, darker distally; fore basitarsus white, 2nd tarsomere white basally, remaining basitarsi cream, darker distally. Scutal chaetotaxy: *np*, 4; *sa*, 1; *pa*, 1; *dc*, 2; *sc*, 1. *Abdomen*. Segments 2–3 orange, rest black, intersegmental membranes white on segments 2–3, silver velutum absent; terminalia dark.

**Figure 9.**
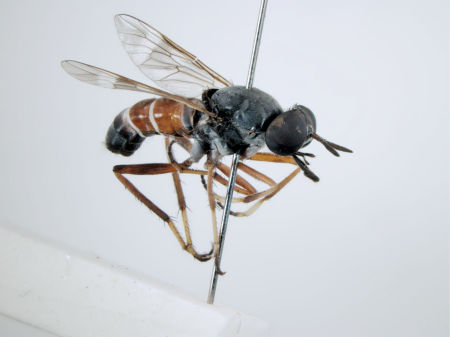
*Acupalpa glossa* sp. n., male, anterolateral view [576249]. Body length = 8.0 mm.

#### Comments.

*Acupalpa glossa* sp. n. is similar to *Acupalpa dolichorhyncha* sp. n. in colour pattern and elongated mouthparts, but is easily differentiated based on leg colour. This species is known only from the type series collected in Victoria.

#### Etymology.

The specific epithet is derived from Gr. *glossa*, tongue, referring to the elongate mouthparts.

### Acupalpa imitans

(White) comb. n.

urn:lsid:zoobank.org:act:749A106A-0357-4AAF-A88B-E2E46ECA7B9A

http://species-id.net/wiki/Acupalpa_imitans

Figs 3C–D, F 10

Phycus imitans White 1915: 28.
Agapophytus imitans (White 1915: 28) - Mann 1929: 40; Irwin and Lyneborg 1989: 354 [catalogue].
Pipinnipons imitans (White 1915: 28) - Winterton et al. 2001: 211.
Acupalpa imitans (White 1915: 28), comb. n.

#### Type material.

**Type** female, AUSTRALIA: **Tasmania:** Wedge Bay, 3.i.1914, G.H. Hardy [lost].

**Neotype** male, AUSTRALIA: **Queensland:** Indooroopilly, Long Pocket [-27.418°, 152.837°], 22.viii–7.ix.2007, S. L. Winterton, Malaise trap (MEI165187) (QM).

#### Other material examined.

AUSTRALIA: **Queensland:** female, Brisbane Forest Park, Scrub Road, crossing at Enoggera Creek, [-27.428°, 152.843°], 200m, 10–14.xi.1995, malaise trap, M.E. Irwin. (MEI140857) (QM); male, Tambourine, [-27.88, 153.13], 12.vi.1925 (“Allotype” of Mann 1929) (MEI023602) (QM); female, Mount Tamborine, [-27.917°, 153.15°], 29.xi.1925, hand netted, H. Hacker (MEI108898) (QM).

#### Diagnosis.

Frons profile rounded above antenna; antenna dark yellow; pleuron black; wing dark banded; femora orange to yellow; tibia yellow; abdomen dark, segments 2–3 red-brown laterally, without silver velutum.

#### Redescription.

Body length= 6.3–7.0 mm. *Head*. Frons wider than ocellar tubercle (female) or narrower (male), profile rounded above antenna, pubescence as silver patches along eye margin, frontal vestiture glabrous, surface texture as irregular longitudinal striations or smooth; face as narrow strip below antennal base, vestiture glabrous; gena with pale setae; parafacial glabrous; mouthparts relatively short (approximately equal to head length); palpus brown-black, narrowly cylindrical; occiput glabrous, glossy black; antennal base flat, frons roughly level with eye in profile (or near so); antenna longer than head; scape dark yellow, length approximately equal to flagellum, scape with sparse black setae; flagellum dark yellow, base of flagellum with short dark setae. *Thorax*. Scutum black, overlain with grey pubescence, brown stripes of pubescence more expansive posteriorly; scutellum overlain with dense, matt-black pubescence; pleuron black, overlain with sparse silver-grey pubescence; wing markings banded infuscate; haltere knob white; coxae black; femora orange or yellow; tibia yellow, apices sometimes dark; tarsi yellow, fore-basitarsus white. Scutal chaetotaxy: *np* 4–5; *sa*, 1; *pa*, 1; *dc*, 2; *sc*, 1. *Abdomen*. Dark, segments 2–3 red-brown medially, orange laterally, silver velutum absent (female) or small triangular patches on tergites 2–3 (male); terminalia pale.

**Figure 10.**
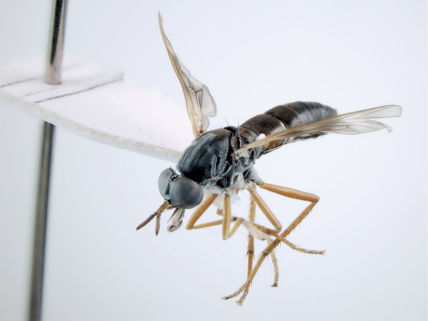
*Acupalpa imitans* (White), male, anterolateral view [576250]. Body length = 7.0 mm.

#### Comments.

The type of *Phycus imitans* was kept in the G.H. Hardy collection, which was moved from Brisbane to Katoomba, but is now apparently lost or destroyed by pests. While not stating whether he actually examined the type, Mann (1929) redescribed this species in *Agapophytus* based on an unjustified allotype designation and associated female from southeast Queensland. Winterton et al. (2001) transferred the species to *Pipinnipons* based on the description of White (1915) and the specimens referred to by Mann (1929). The two specimens examined by Mann (1929) as putative *Acupalpa imitans* were located in the QM collection and no further material has been collected from Tasmania. Based on the discovery of new material from southeast Queensland more closely matching the original description by White (1915) than any other material examined (including the allotype designated by Mann (1929)), a male specimen (MEI165187) is herein designated as a Neotype to stabilise the concept of the species. This problematic species has characteristics that indicate a closer relationship to species of *Acupalpa* (particularly palpi shape) and is herein transferred from *Pipinnipons*.

### Acupalpa irwini

Winterton

urn:lsid:zoobank.org:act:6C3A49B8-6E77-4C55-B5D9-C77636280ECB

http://species-id.net/wiki/Acupalpa_irwini

Fig. 11

Acupalpa irwini Winterton 2000: 232.

#### Type material.

**Holotype** female, AUSTRALIA: **Western Australia:** 7.5 km WSW Lake Cronin, 32°23’S, 119°46’E, 19–26.ix.1978, T. F. Houston et al. (MEI029876) (WAM). **Paratypes**. AUSTRALIA: **Western Australia:** female, same data as holotype, (MEI029877) (WAM); male, 3 females, 53 km E Hyden nr. Emu Rock, 24–27.x.1985, R. W. Thorpe (MEI029502, 029503, 029504, 029505) (UCDC).

#### Diagnosis.

Frons profile rounded above antenna; face projecting anteriorly; antenna black; scutum grey to black; pleuron black; wing dark banded; femora brown to black; abdomen black, segments 1–3 orange; abdominal velutum absent.

#### Redescription.

Body length= 7.0–10.0 mm. *Head*. Frons wider than ocellar tubercle, profile rounded above antenna, pubescence sparse silver-grey, frontal vesiture small dark setae, surface texture verrucous; face projecting anteriorly, vestiture with dark or pale setae; gena with pale setae; parafacial glabrous or with pale setae; mouthparts elongate, projecting anteriorly; palpus brown-black, narrowly cylindrical; occiput overlain with sparse, silver-grey pubescence; antennal base raised; antennal length approximately equal to head; scape black, length approximately equal to flagellum, scape with pale setae ventrally, shorter dark setae dorsally; flagellum black, base of flagellum with short dark setae. *Thorax*. Scutum light grey to black, setal bases glossy black; scutellum overlain with dense, matt-black pubescence; pleuron black, overlain with silver-grey pubescence; wing markings dark banded infuscate; haltere knob brown; coxae black; femora brown to black; tibia yellow-orange, darker distally; tarsi black, basitarsi pale, dark distally. Scutal chaetotaxy: *np*, 4; *sa*, 1; *pa*, 1; *dc*, 2–3; *sc*, 1. *Abdomen*. Segments 1–3 orange, remaining segments black, silver velutum absent; terminalia dark.

**Figure 11.**
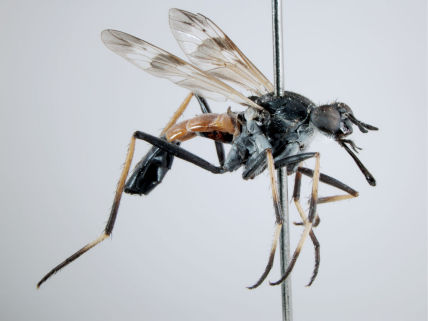
*Acupalpa irwini* Winterton, female, anterolateral view [576251]. Body length = 9.0 mm.

#### Comments.

*Acupalpa irwini* is a relatively large species differentiated by the characteristic leg and abdomen colouration. This species is known only from the type series collected in Western Australia.

### Acupalpa melanophaeos sp. n.

urn:lsid:zoobank.org:act:32E2D131-5108-46B5-B330-ABEAAC192948

http://species-id.net/wiki/Acupalpa_melanophaeos

Figs 3H 12

#### Type material.

**Holotype** female, AUSTRALIA: **Western Australia:** Drummond Cove, Geraldton, 16.xi.1973, N. McFarland [-28.767°, 114.617°] (MEI029498) (ANIC). **Paratypes**. AUSTRALIA: **Western Australia:** female, same data as holotype (MEI029496) (WAM); male, Bunbury, 3.i.1957, A. Snell [-33.317°, 115.633°] (MEI029509) (ANIC); female, Cape Le Grand Nat. Park [-33.96°, 122.12°], 12.i.1987, G. & A. Daniels (MEI029494) (GDCB/AMS).

#### Diagnosis.

Frons profile rounded above antenna; scape yellow, flagellum dark; scutum grey to black; pleuron black (reddish posteriorly in female); wing banded infuscate; coxae orange; legs orange to yellow, tarsi dark distally and fore-basitarsus white; abdomen black, segments 1–3 orange to yellow; abdominal velutum absent.

#### Description.

Body length= 7.1–9.6 mm. *Head*. Frons wider than ocellar tubercle (female) or narrower (male), profile rounded above antenna, pubescence absent, frontal vestiture glabrous or with minute setae, surface texture smooth; face shape broadly rounded, expansive, vestiture glabrous; gena with dark setae; parafacial glabrous; mouthparts elongate, projecting anteriorly, or sometimes relatively short; palpus brown-black, narrowly cylindrical; occiput glabrous, glossy black; antennal base raised (male) or flat, frons roughly level with eye in profile (female); antenna longer than head; scape yellow, length much shorter than flagellum, scape with short, black setae; flagellum black, base of flagellum with short, dark setae. *Thorax*. Scutum light grey to black, setal bases glossy black; scutellum overlain with dense, matt-black pubescence; pleuron black (male) or darker anteriorly with dark orange posteroventrally (female), overlain with sparse silver-grey pubescence; wing markings banded infuscate; haltere knob brown; coxae orange; femora orange or dark yellow; tibia orange; tarsi yellow orange, dark distally, fore-basitarsus white. Scutal chaetotaxy: *np*, 4; *sa*, 1; *pa*, 1; *dc*, 1–2; *sc*, 1. *Abdomen*. Segments 1–3 yellow or orange, remaining segments black, silver velutum absent; terminalia dark.

**Figure 12.**
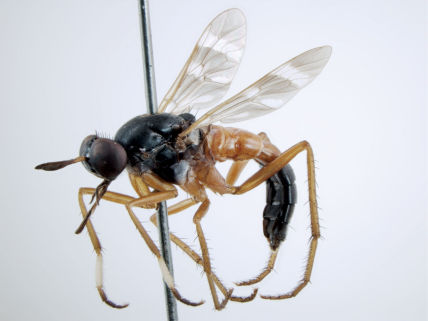
*Acupalpa melanophaeos* sp. n., female, anterolateral view [576252]. Body length = 9.0 mm.

#### Comments.

*Acupalpa melanophaeos* sp. n.is a relatively large species from Western Australia with distinctive leg and abdomen colouration. It is morphologically similar to *Acupalpa novayamarna* sp. n. and *Acupalpa notomelas* sp. n. The coxae are pale in this species along with a rounded face, rather than protruding anteriorly in similar species (e.g. *Acupalpa irwini*).

#### Etymology.

The specific epithet is derived from theGr. *melanos*, black; *phaeos*, light, shiny, referring to the scutum colouration.

### Acupalpa miaboolya sp. n.

urn:lsid:zoobank.org:act:31A86965-E8AB-4D6C-9252-FE42BAC3269A

http://species-id.net/wiki/Acupalpa_miaboolya

Fig. 13

#### Type material.

**Holotype** male, AUSTRALIA: **Western Australia:** 14.5 km N Carnarvon, Miaboolya Beach, [-24.76°, 113.65°], 4.x.1969, H. E. Evans, R. W. Matthews. (MEI080305) (ANIC). **Paratype**. AUSTRALIA: **Western Australia:** female, same data as holotype (MEI080301) (MCZ).

#### Diagnosis.

Frons profile rounded above antenna; antenna brown to black; scutum glossy black with pubescent stripes of grey and brown; pleuron black; wing faintly infuscate; femora and tibia dark, fore tibia pale distally; abdomen black, without silver velutum.

#### Description.

Body length= 5.0–6.5 mm. *Head*. Frons wider than ocellar tubercle, profile rounded above antenna, pubescence sparse silver-grey, without setae, surface texture smooth; face broadly rounded, glabrous; gena with pale setae (female) or dark setae (male); parafacial glabrous; mouthparts relatively short (approximately equal to head length), or elongate and projecting anteriorly; palpus brown-black, acuminate; occiput glabrous, glossy black; antennal base flat; frons roughly level with eye in profile; antennal length approximately equal to head; scape light brown to black, length approximately equal to flagellum, scape with sparse black setae; flagellum black, base of flagellum with short, dark setae. *Thorax*. Scutum black, overlain with stripes of grey and brown pubescence; scutellum overlain with sparse grey pubescence; pleuron black, overlain with sparse silver-grey pubescence; wing largely hyaline, faint band midway (male) or infuscate with pale band midway, hyaline ocellations basally (female); haltere knob white; coxae black, overlain with silver pubescence; femora brown to black; tibia brown; fore tibia pale distally; tarsi black, mid and hind basitarsi pale basally. Scutal chaetotaxy: *np*, 3; *sa*, 1; *pa*, 1; *dc*, 3; *sc*, 1. *Abdomen*. Black, overlain with bronze pubescence, silver velutum absent; terminalia dark.

**Figure 13.**
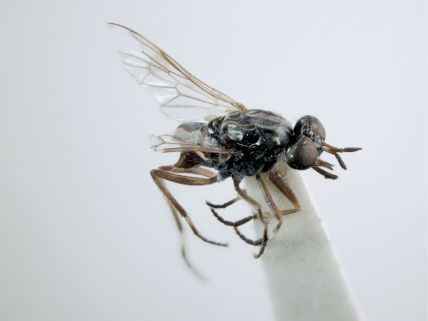
*Acupalpa miaboolya* sp. n., male, anterolateral view [576253]. Body length = 5.0 mm.

#### Comments.

*Acupalpa miaboolya* sp. n. is a relatively small, dark species from Western Australia very similar to *Acupalpa boharti* sp. n. This species can be differentiated from the latter based on scutal pattern; the scutum has grey and brown stripes in *Acupalpa miaboolya* sp. n. while the scutum of *Acupalpa boharti* sp. n. has more uniform brown-bronze pubescence.

#### Etymology.

This species is named after the region in which the specimens were collected, Miaboolya beach, on the north-central coast of Western Australia.

### Acupalpa minuta sp. n.

urn:lsid:zoobank.org:act:C2B0BF78-A250-4F9E-B23C-44533147EFA5

http://species-id.net/wiki/Acupalpa_minuta

Fig. 14

#### Type material.

**Holotype** male, AUSTRALIA: **Western Australia:** Kalbarri, [-27.717, 114.167], 23.ix.1974, N. McFarland (MEI021410) (ANIC).

#### Diagnosis.

Very small sized species; setae on coxae pale; flagellum greatly elongate; scape relatively short; frons equal to width of ocellar tubercle; tibia dark; two notopleural setae; abdomen dark, velutum absent.

#### Description.

Body length= 3.0 mm. *Head*. Frons wider than ocellar tubercle, profile rounded, level with eye, pubescence sparse silver-grey; frontal vestiture glabrous, texture smooth; lower frons and face broadly rounded, expansive; face vestiture glabrous; gena with pale setae; parafacia overlain with silver pubescence; mouthparts elongate, projecting anteriorly; palpus brown-black; occiput overlain with sparse, silver-grey pubescence; antennal base flat; antennal length longer than head; scape colour black, length much shorter than flagellum, with sparse black setae; flagellum colour brown, base of flagellum without setae. *Thorax*. Scutum glossy black-brown with sparse grey pubescence and small brown setae; scutellum overlain with dense matt black pubescence; pleuron glossy black-brown with longitudinal stripe of silver velutum; wing markings dark banded infuscate; haltere knob orange-yellow; coxae brown, overlain with dense pubescence and pale setae; femora brown to black; tibia brown; tarsi brown. Scutal chaetotaxy (macrosetae pairs): *np*, 2; *sa*, 1; *pa*, 1; *dc*, 3, *sc*, 1. *Abdomen*. Colouration brown, tergites 2-4 with bronze pubescence, silver velutum absent; terminalia dark.

**Figure 14.**
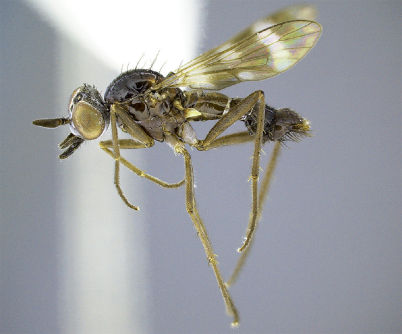
*Acupalpa minuta* sp. n., male, lateral view [581505]. Body length = 3.0 mm.

#### Comments.

*Acupalpa minuta* sp. n. is closely related to *Acupalpa minutoides* sp. n., sharing characteristics such as very small size, two notopleural setae and an antenna with a short scape and a greatly elongate flagellum. It can be differentiated based on the colour of the setae on the coxae and by the width of the frons. This species is known only from a single male individual from Western Australia.

#### Etymology.

The specific epithet is derived from the L. *minutus*, small, little, referring to the diminutive body size.

### Acupalpa minutoides sp. n.

urn:lsid:zoobank.org:act:2429322A-907F-4330-8BF5-0F1C34FBFAD7

http://species-id.net/wiki/Acupalpa_minutoides

Fig. 15

#### Type material.

**Holotype** male, AUSTRALIA: **Western Australia:** Geraldton, Drummond’s Cove, [-28.767, 114.617], 29.ix.1972, N. McFarland (MEI021412) (ANIC). **Paratypes**. **Western Australia:** male, Geraldton, Drummond’s Cove, [-28.767, 114.617], 29.ix.1972, N. McFarland, on *Calandrinia* flowers (MEI029995) (CSCA); male, Geraldton, Drummond’s Cove, [-28.767, 114.617], 18.ix.1972, N. McFarland, on *Calandrinia* flowers (MEI021411) (CSCA).

#### Diagnosis.

Very small sized species; setae on coxae black; flagellum greatly elongate; scape relatively short; frons slightly wider than width of ocellar tubercle; wing veins M1 and M2 fused and petiolate basally from discal cell; tibia pale basally; two notopleural setae; abdomen dark, velutum absent.

#### Description.

Body length= 3.0–4.0 mm. *Head*. Frons wider than ocellar tubercle, profile rounded, level with eye, pubescence sparse silver-grey; frontal vestiture glabrous with minute setae laterally, texture smooth; lower frons and face shape broadly rounded, expansive; face vestiture glabrous; gena with pale setae; parafacia overlain with silver pubescence; mouthparts elongate, projecting anteriorly; palpus brown-black; occiput overlain with sparse, silver-grey pubescence; antennal base flat; antennal length longer than head; scape black, length much shorter than flagellum, with sparse black setae; flagellum black or brown, base of flagellum without setae. *Thorax*. Scutum glossy black-brown with sparse grey pubescence and small brown setae; scutellum overlain with dense matt black pubescence; pleuron glossy black-brown with longitudinal stripe of silver velutum; wing markings dark banded infuscate; haltere knob orange-yellow; coxae brown, overlain with dense pubescence and dark setae; femora brown to black; tibia black, yellow-orange basally; tarsi brown. Scutal chaetotaxy (macrosetae pairs): *np*, 2, *sa*, 1, *pa*, 1, *dc*, 2, *sc*, 1. *Abdomen*. Colouration brown, tergites 2-4 with bronze pubescence, silver velutum absent; terminalia dark.

**Figure 15.**
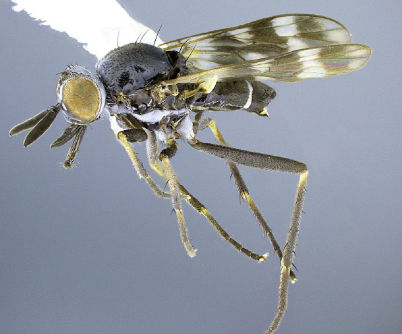
*Acupalpa minutoides* sp. n., male, lateral view [581506]. Body length = 3.5 mm.

#### Comments.

See comments under *Acupalpa minuta* sp. n. This species is known only from three male specimens from Western Australia.

#### Etymology.

The specific epithet is derived from the L. *minutus*, small, little; -*oides*, like, referring to the similarity of this species to *Acupalpa minuta* sp. n.

### Acupalpa notomelas sp. n.

urn:lsid:zoobank.org:act:68193274-CA84-43A8-A35A-6E819DE50B46

http://species-id.net/wiki/Acupalpa_notomelas

Figs 3A–B 16

#### Type material.

**Holotype** male, AUSTRALIA: **Western Australia:** 22 km W Waroora Homestead [-23.483°, 113.8°], 25.x.1987, sand plain, M. E. Irwin (MEI029510) (ANIC). **Paratypes**. AUSTRALIA: **Western Australia:** female, Melaleuca Park, 38 km N Perth [-31.95°, 115.85°], 29.x.1987, M. E. Irwin, E. I. Schlinger (MEI029512) (ANIC); male, Yanchep National Park [-31.517°, 115.683°], 22–26.x.1985, truck trap, A. Dyce, W. Wirth (MEI029514) (ANIC).

#### Diagnosis.

Frons profile rounded above antenna; mouthparts elongate; antenna dark; scutum dark; pleuron orange ventrally; wing banded; legs dark yellow to orange [fore femur darker]; abdomen black without silver velutum.

#### Description.

Body length= 6.2–8.0 mm. *Head*. Frons wider than ocellar tubercle, profile rounded above antenna, glabrous or silver pubescent patches along eye margin [some sparse pubescence dorsally], frontal vestiture as small dark setae, surface texture smooth, face broadly rounded, vestiture as dark or pale setae; gena with pale setae or with dark setae (ventrally); parafacial glabrous; mouthparts elongate, projecting anteriorly; palpus brown-black, narrowly cylindrical; occiput overlain with sparse, silver-grey pubescence, antennal base flat; antennal length approximately equal to head; scape brown, length shorter than flagellum, scape with sparse black setae; flagellum dark, base of flagellum without setae. *Thorax*. Scutum light grey to glossy black, setal bases glossy black, overlain with faint stripes of grey pubescence; scutellum overlain with dense, matt-black pubescence; pleuron yellow-orange in lower 2/3, upper 1/3 concolourous with scutum, overlain with sparse silver-grey pubescence; wing markings banded infuscate; haltere knob white, dark basally; coxae yellow-orange; femora with darker fore femur, rest yellow-orange; tibia and tarsi dark yellow; fore-basitarsus white, darker basally, 2nd tarsomere white basally, remaining basitarsi yellow-brown. Scutal chaetotaxy: *np*, 4; *sa*, 1; *pa*, 1; *dc*, 2; *sc*, 1. *Abdomen*. black, silver velutum absent; terminalia pale.

**Figure 16.**
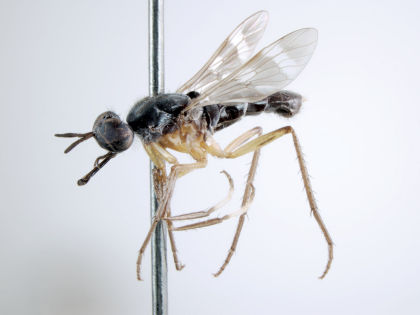
*Acupalpa notomelas* sp. n., male, anterolateral view [576254]. Body length = 6.0 mm.

#### Comments.

*Acupalpa notomelas* sp. n. is similar to *Acupalpa melanophaeos* sp. n. and *Acupalpa novayamarna* sp. n., but can be differentiated based on abdomen colouration. The palpi are very slender and elongate.

#### Etymology.

The specific epithet is derived from the Gr. *noto*, back; *melas* black, dark, referring to the scutal colouration.

### Acupalpa novayamarna sp. n.

urn:lsid:zoobank.org:act:C0BEA372-6953-43E4-B957-0A521FD8D83B

http://species-id.net/wiki/Acupalpa_novayamarna

Fig. 17

#### Type material.

**Holotype** male, AUSTRALIA: **Western Australia:** 25 km E New Yamarna Homestead, [-28.167°, 123.683°], 21.ix.1982, T. F. Houston, B. Hanich (WAM872128) (WAM).

#### Diagnosis.

Frons profile rounded to slightly concave above antenna; mouthparts short; antenna dark; scutum dark; pleuron dark dorsally, orange ventrally; wing banded; legs orange, tarsi dark distally, fore-basitarsus white; abdomen orange, segments 1–2 black medially, velutum absent.

#### Description.

Body length= 7.2 mm. *Head*. Frons wider than ocellar tubercle, profile rounded to slightly concave above antenna, pubescence absent or as silver patches along eye margin, frontal vestiture glabrous, surface texture smooth, face as narrow strip below antennal base, glabrous; gena with pale setae; parafacial overlain with silver pubescence; mouthparts relatively short (approximately equal to head length); palpus brown-black, narrowly cylindrical; occiput glabrous, glossy black; antennal base raised, antennal length approximately equal to head; scape orange-brown, length much shorter than flagellum, scape with sparse black setae; flagellum black, base of flagellum with short dark setae. *Thorax*. Scutum light grey to black, setal bases glossy black; scutellum overlain with dense, matt-black pubescence; pleuron orange, upper 1/3 concolourous with scutum, overlain with sparse silver-grey pubescence; wing markings banded infuscate, dark yellow basally; haltere knob orange-yellow; coxae orange; femora orange; tibia orange; tarsi yellow orange, dark distally, fore-basitarsus white. Scutal chaetotaxy: *np*, 4; *sa*, 1; *pa*, 1; *dc*, 2; *sc*, 1. *Abdomen*. Orange, segments 1–2 black medially, silver velutum absent; terminalia pale.

**Figure 17.**
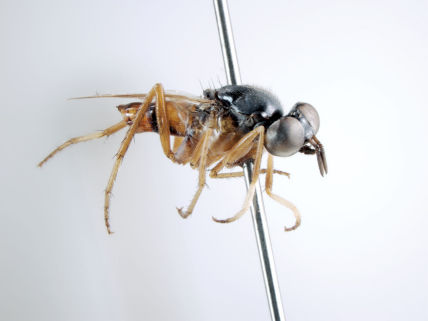
*Acupalpa novayamarna* sp. n., male, anterolateral view [576255]. Body length = 7.0 mm.

#### Comments.

This species is very similar to *Acupalpa melanophaeos* sp. n., but can be differentiated by the pale genal setae, larger proportion of the pleuron being orange, and male frons slightly wider. Only the male is known for this western species.

#### Etymology.

This species is named after the type locality of New Yamarna Homestead, Western Australia.

### Acupalpa rostrata

Kröber

urn:lsid:zoobank.org:act:4FFB52C9-C88E-426B-BB46-B2CF1EC04C9F

http://species-id.net/wiki/Acupalpa_rostrata

Figs 3E, G, I 18

Acupalpa rostrata Kröber 1912: 152; Kröber 1913: 18; Mann 1929: 26; Irwin and Lyneborg 1989: 354 [catalogue]; Winterton 2000: 235; Winterton et al. 2001: 210.

#### Type material.

**Holotype** male, AUSTRALIA: **New South Wales**, Sydney (ZMUH) [destroyed]. **Neotype** male, AUSTRALIA: **New South Wales:** Kosciusko National Park, Round Mountain, Olgives Creek, [-35.682°, 149.533°] 1400m, 28.xii.1977, E. I. Schlinger. (MEI029931) (ANIC).

#### Diagnosis.

Frons profile concave above antenna; scape and pedicel yellow-orange, flagellum black; scutum black, overlain with silver pubescence; pleuron black with silver pubescence; wing banded; femora yellow-orange [hind femur dark]; tibia yellow-orange; abdomen black, overlain with silver velutum in male.

#### Redescription.

Body length= 7.0–8.0 mm. *Head*. Frons wider than ocellar tubercle, profile transversely concave above antennae, pubescence as silver patches along eye margin, frontal vestiture as numerous elongate setae, surface texture smooth; face projecting anteriorly, vestiture as dark or pale setae; gena with pale setae; parafacial glabrous; mouthparts relatively short (approximately equal to head length); palpus brown-black, acuminate; occiput glabrous, glossy black; antennal base raised; antennal length approximately equal to head; scape and pedicel yellow-orange, length approximately equal to flagellum, with pale setae ventrally, shorter dark setae dorsally; flagellum black, base of flagellum with short dark setae. *Thorax*. Scutum uniform grey-black; scutellum overlain with dense, matt-black pubescence; pleuron black, overlain with silver pubescence; wing markings banded infuscate; haltere knob white, dark basally; coxae black; femora yellow with hind femur dark; tibia yellow-orange, apices dark; tarsi yellow-orange, distal segments darker, basitarsus and second tarsomere on foreleg white. Scutal chaetotaxy: *np*, 4; *sa*, 1; *pa*, 1; *dc*, 2; *sc*, 1. *Abdomen*. Black, silver velutum dorsally on tergites (male) or absent (female); terminalia pale.

**Figure 18.**
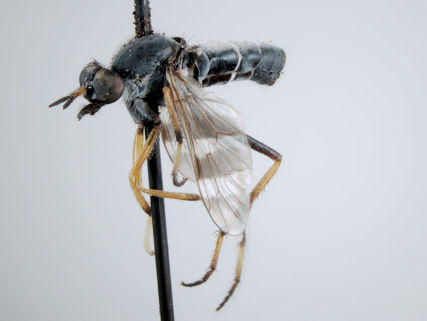
*Acupalpa rostrata* Kröber, Neotype male, anterolateral view [576256]. Body length = 7.0 mm.

#### Comments.

The type of *Acupalpa rostrata* is apparently destroyed. Winterton (2000) redescribed this distinctive species, without designating a neotype as the species was still identifiable based on the original description alone. With the description of the new species herein a neotype is designated to stabilise the taxon and remove any possibility of confusion in the future. *Acupalpa rostrata* is differentiated from other *Acupalpa* species by the unique leg and antennal colouration.

### Acupalpa semirufa

Mann

urn:lsid:zoobank.org:act:CF947CED-BCB0-4A62-AFDF-433DDBF3E1D3

http://species-id.net/wiki/Acupalpa_semirufa

Fig. 19

Acupalpa semirufa Mann 1929: 27; Irwin and Lyneborg 1989: 354 [catalogue]; Winterton 2000: 237; Winterton et al. 2001: 210.

#### Type material.

**Holotype** male, AUSTRALIA: **New South Wales:** Blackheath, Hardy[not examined-location unknown]. **Paratypes**. AUSTRALIA: **Queensland**: **‘**Allotype’ female, Bribie Island, 12.ix.1918, H. Hacker (MEI029439) (QM). **New South Wales:** 2 females, Sydney, Manly, 20.xi.1923 (MEI108786, 108787) (QM).

#### Diagnosis.

Frons profile concave above antenna; antenna dark; scutum dark; pleuron black; wing dark banded; femora orange to yellow; tibia yellow, darker distally; abdomen black with segments 2–3 yellow-orange, overlain with silver velutum in male.

#### Redescription.

Body length= 6.0–9.0 mm. *Head*. Frons wider than ocellar tubercle, profile transversely concave above antennae, pubescence as silver patches along eye margin, sparse silver-grey dorsally, frontal vestiture as small dark setae, surface texture verrucous; face projecting anteriorly with dark or pale setae; gena with pale setae; parafacial glabrous; mouthparts short (approximately equal to head length), or elongate, projecting anteriorly; palpus brown-black, acuminate; occiput glabrous, glossy black; antennal base raised; antennal length approximately equal to head; scape brown, length shorter than flagellum, scape with pale setae ventrally, shorter dark setae dorsally; flagellum black, base of flagellum with short dark setae. *Thorax*. Scutum uniform grey-black or light grey to black, setal bases glossy black; scutellum overlain with dense, matt-black pubescence; pleuron black, overlain with sparse silver-grey pubescence; wing markings dark, banded infuscate; haltere knob white, dark basally; coxae black; femora orange or yellow; tibia yellow-orange, darker distally; basitarsi yellow-orange, rest black, fore-basitarsus white distally, 2nd tarsomere basally. Scutal chaetotaxy: *np*, 4; *sa*, 1; *pa*, 1; *dc*, 3; *sc*, 1. *Abdomen*. Segment 2 in male, or segments 2–3 in female orange with black medial patch, rest of segments black, silver velutum dorsally on tergites (male) or absent (female); terminalia pale.

**Figure 19.**
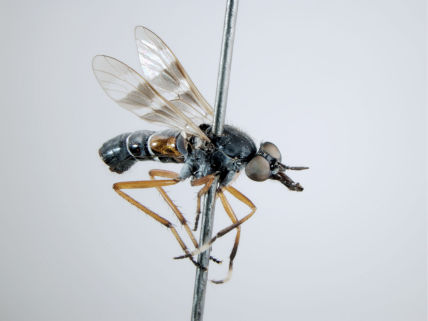
*Acupalpa semirufa* Mann, male, anterolateral view [576257]. Body length = 7.0 mm.

#### Comments.

*Acupalpa semirufa* is a common species in south-eastern Queensland and northern New South Wales. It is similar to *Acupalpa divisa* and *Acupalpa yalgoo* sp. n., but can be differentiated easily from the former by the orange leg colour (black in *Acupalpa divisa*) and from the latter by the projecting face (rounded in *Acupalpa yalgoo* sp. n.) and two wing bands (single band in *Acupalpa yalgoo* sp. n.).

### Acupalpa westralica sp. n.

urn:lsid:zoobank.org:act:C2BCA147-940D-42D1-AB6C-0A106BF6709A

http://species-id.net/wiki/Acupalpa_westralica

Fig. 20

#### Type material.

**Holotype** female, AUSTRALIA: **Western Australia:** Stirling Ranges N.P., Chester Pass Rd., Eucalyptus open woodland, 230m; C. Lambkin, J. Recsei, 3–15.xi.2003; Malaise, ANIC bulk sample 2191 [-34.433°, 118.076°] (MEI165188) (ANIC).

#### Diagnosis.

Frons profile rounded above antenna; scape yellow-brown, flagellum black; scutum grey-black with dark and pale stripes; pleuron black; wing dark banded; femora yellow with extensive dark suffusion dorsally [hind femur dark]; tibia brown; abdomen black with brown pubescence, silver velutum absent.

#### Description.

Body length= 8.0 mm. *Head*. Frons wider than ocellar tubercle, profile rounded above antenna, glabrous or with minute setae, surface texture smooth; face broadly rounded, expansive, with dark or pale setae; gena with pale setae; parafacial glabrous; mouthparts elongate, projecting anteriorly; palpus brown-black, narrowly cylindrical; occiput glabrous, glossy black; antennal base flat; frons roughly level with eye in profile; antennal length approximately equal to head; scape yellow-brown, shorter than flagellum, scape with sparse black setae; flagellum black, base of flagellum without setae. *Thorax*. Scutum uniform grey-black with diffuse brown and cream stripes; scutellum overlain with dense, matt-black pubescence; pleuron black, overlain with sparse silver-grey pubescence; wing markings dark banded infuscate; haltere knob white, dark basally; coxae yellow; hind femur dark, rest yellow with extensive dark suffusion dorsally; tibia and tarsi brown, fore-basitarsus white. Scutal chaetotaxy: *np*, 4; *sa*, 1; *pa*, 1; *dc*, 2; *sc*, 1. Abdomen. Black with brown pubescence, silver velutum absent; terminalia dark.

**Figure 20.**
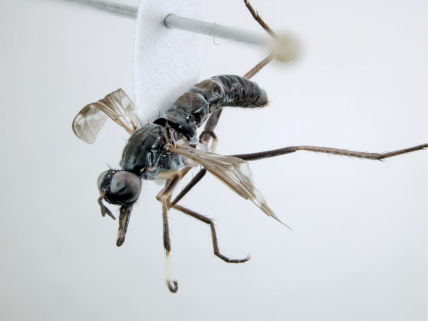
*Acupalpa westralica* sp. n., female, anterolateral view [576258]. Body length = 8.0 mm.

#### Comments.

*Acupalpa westralica* sp. n. is known only from the holotype female from southern Western Australia. This species superficially resembles *Acupalpa rostrata* in colouration, but the head shape suggests a close relationship to *Acupalpa notomelas* sp. n.

#### Etymology.

The specific epithet is derived from the western distribution of this species.

### Acupalpa yalgoo sp. n.

urn:lsid:zoobank.org:act:AA49E372-62DF-4607-9C4E-77CCE53D9CC4

http://species-id.net/wiki/Acupalpa_yalgoo

Fig. 21

#### Type material.

**Holotype** male, AUSTRALIA: **Western Australia**, 28 km W Yalgoo, [-28.35°, 116.683°], 2.ix.1981, G. A. Holloway (MEI029508 ) (AM). **Paratype**. AUSTRALIA: **Western Australia:** male, Great Victoria Desert, Officer Basin, NE Streich Mound, 24–28.ix.1991, McMillan (MEI165193) (WAM).

#### Diagnosis.

Frons profile rounded above antenna; antenna black; scutum grey to black; pleuron black; wing dark banded; femora black; tibia yellow in basal half; abdomen black, segments 1–3 orange, silver velutum absent.

#### Description.

Body length= 9.0–10.0 mm. *Head*. Frons wider than ocellar tubercle, profile rounded above antenna, pubescence sparse silver-grey, frontal vestiture as small dark setae, surface texture as irregular longitudinal striations, face broadly rounded, expansive, with dark or pale setae; gena with pale setae; parafacial with short setae towards gena; mouthparts elongate; palpus brown-black, narrowly cylindrical; occiput overlain with sparse, silver-grey pubescence; antennal base flat; frons roughly level with eye in profile; antennal length shorter than head; scape black, length approximately equal to flagellum, scape with sparse black setae; flagellum black. *Thorax*. Scutum light grey to black, setal bases glossy black; scutellum overlain with dense, matt-black pubescence; pleuron black, overlain with sparse silver-grey pubescence; wing markings dark banded [discal band broad], dark yellow basally; haltere knob orange-yellow; coxae black; femora brown to black; tibia yellow in basal 1/2, dark in distal 1/2; tarsi black, basitarsi pale, dark distally, rest of tarsomeres dark. Scutal chaetotaxy: *np*, 4–5; *sa*, 1; *pa*, 1; *dc*, 3; *sc*, 2. *Abdomen*. Segments 1–3 orange, remaining segments black, silver velutum absent; terminalia dark.

**Figure 21.**
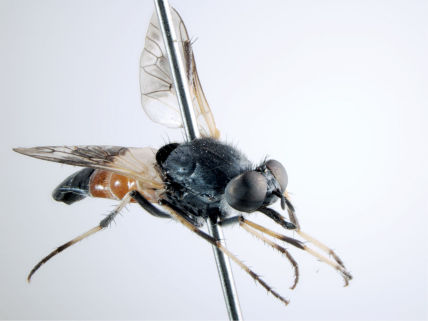
*Acupalpa yalgoo* sp. n., male, anterolateral view [576259]. Body length = 8.0 mm.

#### Comments.

*Acupalpa yalgoo* sp. n. is a western species similar to *Acupalpa semirufa* and *Acupalpa glossa* sp. n., but differs from both in leg colouration and wing patterning.

#### Etymology.

The specific epithet is derived from the type locality of this species, near the Western Australian township of Yalgoo.

### Acupalpa yanchep sp. n.

urn:lsid:zoobank.org:act:A3312038-6598-4C6A-BBA4-09CFF0E002F0

http://species-id.net/wiki/Acupalpa_yanchep

Figs 2 22

#### Type material.

**Holotype** male, AUSTRALIA: **Western Australia:** Yanchep N.P., Malaise trap, [-31.576°, 115.693°] 18–29.xii.1999; J. & A. Skevington, C. Lambkin, P. Bouchard (MEI165189) (ANIC). **Paratypes**. AUSTRALIA: **Western Australia:** male, female, same data as holotype (MEI165190, 165191) (ANIC); female, Yanchep [-31.525°, 115.626°], 21.xi.2008, fore-dune, S. L. Winterton & S. D. Gaimari (MEI165192) (QM).

#### Diagnosis.

Frons profile rounded above antenna; antenna dark; scutum black with irregular brown and white pubescent markings; pleuron black; wing irregularly banded; legs dark, tibia pale basally; abdomen black [sometimes orange apically in female], silver velutum present in male.

#### Description.

Body length= 6.0–10.0 mm. *Head*. Frons wider than ocellar tubercle (female) or narrower (male), profile rounded above antenna (male) or transversely concave above antennae (female), pubescence as silver patches along eye margin, frontal vestiture as small dark setae, surface texture as irregular longitudinal striations (female) or smooth (male); face broadly rounded with dark or pale setae; gena with pale setae; parafacial overlain with silver pubescence; mouthparts length variable, but usually relatively short; palpus brown-black, acuminate; occiput overlain with sparse, silver-grey pubescence; antennal base raised; antenna longer than head; scape brown, length approximately equal to flagellum, scape with sparse black setae; flagellum black, base of flagellum with short, dark setae. *Thorax*. Scutum dark, overlain with pubescence of irregular brown to grey markings with pale broken lines and spots, setal bases glossy black; scutellum overlain with grey and matte black pubescence; pleuron dark, overlain with silver-grey pubescence; wing markings irregularly banded to apparently fenestrate; haltere knob white; coxae black, overlain with silver pubescence; femora brown to black; tibia black, yellow-orange dorsal stripe in basal 1/2; tarsi white, basitarsi dark in basal 3/4. Scutal chaetotaxy: *np*, 3–5; *sa*, 1 [rarely 2–3]; *pa*, 1; *dc*, 2–3; *sc*, 1. *Abdomen*. Black (segments 6-8 orange in some females), silver velutum dorsally on tergites (male) or absent (female); terminalia pale.

**Figure 22.**
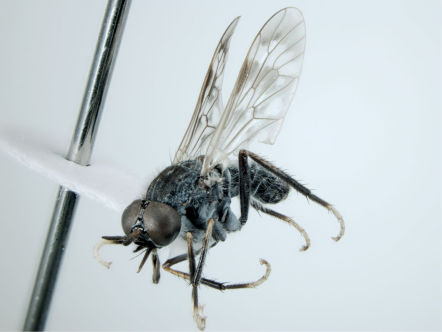
*Acupalpa yanchep* sp. n., male, anterolateral view [576260]. Body length = 6.0 mm.

#### Comments.

*Acupalpa yanchep* sp. n. is morphologically similar to *Acupalpa albitarsa*, with females difficult to separate except for more pronounced white scutal patterning in many individuals. This species is found in western Australia while *Acupalpa albitarsa* is found in eastern and southern regions. There is a pronounced size difference in the sexes of *Acupalpa yanchep* sp. n. with males considerably smaller than females. The leg colouration and scutal patterning is distinctive for this species.

#### Etymology.

This species is named after the type locality, the township of Yanchep, Western Australia.

### Pipinnipons

Winterton

urn:lsid:zoobank.org:act:157A683F-6C11-4309-A7F5-5B5C4C37E47C

http://species-id.net/wiki/Pipinnipons

Pipinnipons Winterton, 2001: 205. Type species: *Pipinnipons kroeberi* Winterton, 2001: 206.

#### Diagnosis.

Antenna elongate, cylindrical, total length slightly longer than or equal to head length; scape shorter than flagellum; frons flat, smooth; face as narrow strip below antenna, glabrous; palpus spatulate apically; mouthparts short; occiput with single row of postocular setae immediately laterad of ocellar tubercle in male, multiple rows in female; wing banded infuscate or hyaline; setae absent on wing vein R1; cell m3 closed; elongate velutum patches on fore and hind femora; femora without macrosetae; single type of setal pile on femora, setae not appressed; prosternal furrow without setae; mid coxa without setae on posterior surface; post spiracular pile absent; gonocoxites with velutum patch on ventral surface (Fig. 24); articulated gonocoxal process present; hypandrium present; ventral apodeme of parameral sheath forked; dorsal apodeme of parameral sheath ‘T’-shaped; three spermathecae in female; spermathecal sac present, usually with two smaller, additional lobes and/or outer reticulated lobes along length; spermathecal ducts joining common duct before bursa; female with A1 and A2 acanthophorite spines well developed; female sternite 8 emarginate along posterior margin.

#### Comments.

**Figure 23.**
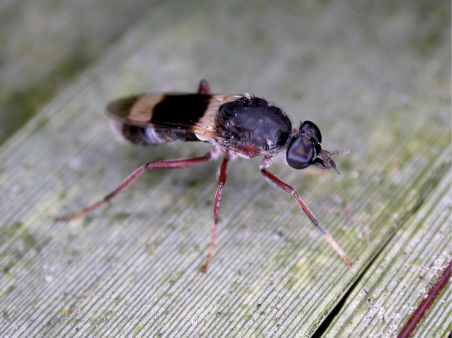
*Pipinnipons fascipennis* (Kröber),male. Body length = 6.0 mm. (Photo: S.L. Winterton).

*Pipinnipons* is a distinctive genus of wasp mimicking therevids, often with metallic pubescence, yellow and black marking and banded wings (Fig. 23). It can be distinguished among related genera by the elongate, cylindrical antennae, scape not longer than flagellum, narrow face and palpi spatulate. The latter two characters specifically differentiate *Pipinnipons* from *Acupalpa*, as the face is broadly rounded, often produced, and the palpi are acuminate or narrowly cylindrical in *Acupalpa*. While the mouthparts are of variable length in *Acupalpa* (and often elongate and forward projecting), the mouthparts of *Pipinnipons* are always relatively short. As stated in the comments under *Acupalpa*, *Agapophytus* is separated from *Pipinnipons* and *Acupalpa* by the length of the scape ranging from relatively equal length, to significantly longer than the flagellum. The modified setae patch on abdominal tergite 2 mentioned by Winterton et al. (2001) as a characteristic of *Pipinnipons* is not present in all the new species described here, and is no longer considered diagnostic for the genus as it is also found sporadically in other, unrelated genera such as *Neodialineura* Mann, 1928 and *Bonjeania* Irwin and Lyneborg, 1989. The male terminalia are relatively conserved throughout the genus, and species identification is more easily done using external characters of both sexes. *Pipinnipons* is distributed along coastal eastern Australia from northern Queensland to Tasmania.

#### Included species.

*Pipinnipons chauncyvallis* sp. n., *Pipinnipons fascipennis* (Kröber), *Pipinnipons kampmeierae* sp. n., *Pipinnipons kroeberi* Winterton, *Pipinnipons sphecoda* sp. n.

#### Key to *Pipinnipons* species:

**Table:** 

1	Abdomen with bright yellow and black markings (Fig. 29)	*Pipinnipons sphecoda* sp. n.
–	Abdomen otherwise coloured and marked	2
2	Abdominal tergites 4–8 overlain with dense silver to gold velutum; legs and pleuron dark orange-maroon (Figs 23^26^)	*Pipinnipons fascipennis* (Krober)
–	Abdominal tergites 4–8 without dense silver to gold velutum; leg colour variable, usually bright orange (Figs 27–28) to yellow with dark markings (Fig. 25); pleuron black	3
3	Legs and coxae brown and yellow; wings banded infuscate; abdomen entirely brown to black (Fig. 25) (Tasmania)	*Pipinnipons chauncyvallis* sp. n.
–	Legs and coxae orange; wings largely hyaline or very weakly banded infuscate; some abdominal segments orange (Queensland)	4
4	Hind coxa dark; abdomen mostly orange (segments 3–4 with black suffusion); intersegmental margin of segment 3 not lighter (Fig. 28)	*Pipinnipons kroeberi* Winterton
–	Hind coxa orange; abdominal segments 2–3 orange with black dorsum, rest glossy black; intersegmental margin of segment 3 white (Fig. 27)	*Pipinnipons kampmeierae* sp. n.

### Pipinnipons chauncyvallis sp. n.

urn:lsid:zoobank.org:act:718CB653-E72C-4333-A6C0-C54249CFDAEE

http://species-id.net/wiki/Pipinnipons_chauncyvallis

Fig. 25

#### Type material.

**Holotype** male, AUSTRALIA: **Tasmania:** Bagdad, Chauncyvale Wildlife Sanctuary [-42.614°, 147.256°], 18–19.xii.1998, D. Yeates, S. Winterton (ANIC29_021139) (ANIC). **Paratypes:** AUSTRALIA: **Tasmania:** 3 females, same data as holotype (ANIC29_021136, 021137, 021140) (ANIC).

#### Diagnosis.

Wing banded; pleuron black; femora yellow [hind femur dark]; tibia yellow, darker apically]; abdomen black, segments 6–8 orange [female].

#### Description.

Body length= 7.0–10.0 mm. *Head*. Frons wider than ocellar tubercle in female, equal in male, profile flat to rounded above antenna, pubescence absent, frontal vestiture as numerous elongate setae (longer in male), surface texture as irregular longitudinal striations or transverse striations; gena with pale setae; parafacial overlain with silver pubescence; palpus yellow-orange; occiput glabrous, glossy black; antennal base raised; antenna longer than head; scape yellow, length shorter than flagellum, with sparse black setae; flagellum dark yellow (darker basally), base of flagellum with short dark setae. *Thorax*. Scutum uniform grey-black with white pile and overlain with sparse grey pubescence; scutellum overlain with dense, matt-black pubescence; pleuron black, overlain with sparse silver-grey pubescence; wing markings banded infuscate; haltere knob white, dark basally; coxae black; hind femur dark, rest yellow with dark patch; tibia yellow (apices sometimes darker); tarsi yellow orange, dark distally, fore-basitarsus white. Scutal chaetotaxy: *np*, 3–4; *sa*, 1; *pa*, 1; *dc*, 2; *sc*, 1. *Abdomen*. Black, segments 6–8 orange in female; terminalia pale.

#### Comments.

*Pipinnipons chauncyvallis* sp. n. is known only from a small conservation area near Bagdad, Tasmania. This species differs from all other *Pipinnipons* by the body colouration and the numerous pale setae on the frons.

#### Etymology.

This species is named after the type locality, Chauncyvale Wildlife Sanctuary, owned by the Chauncy family who established and maintain the sanctuary.

### Pipinnipons fascipennis

(Kröber)

urn:lsid:zoobank.org:act:336E5D1F-DAAF-4532-BFFB-1CAFD12670E8

Genbank Accession: AF150979

http://species-id.net/wiki/Pipinnipons_fascipennis

Fig. 23 24A–D, G 26

Squamopygia fascipennis Kröber 1928: 36.
Pipinnipons fascipennis (Kröber) - Winterton et al. 2001: 211.

#### Type material.

**Type** male. AUSTRALIA: **Queensland:** Kuranda [-16.817°, 145.633°], Lichtwardt (MEI090896) (DEI).

*Other material examined-* AUSTRALIA: **Queensland:** male, Indooroopilly, Long Pocket, 22.viii–7.ix.2007, S. L. Winterton, Malaise trap (UQIC) (MEI165213).

#### Diagnosis.

Wing dark banded; pleuron orange to maroon; legs orange to maroon, tarsi lighter; abdomen dark red, tergite 2–3 red-brown, gold-bronze velutum on segments 4–7.

#### Redescription.

Body length= 7.0–9.0 mm. *Head*. Frons wider than ocellar tubercle (female) or narrower (male), profile rounded above antenna, pubescence matte black and bronze, surface texture smooth or striated; gena with pale setae; parafacial overlain with silver pubescence; palpus brown-black; occiput glabrous, glossy black; antennal base raised; antennal length approximately equal to head; scape orange to brown, shorter than flagellum, with sparse black setae; flagellum orange to brown, base of flagellum with short dark setae. *Thorax*. Scutum dark with irregular brown and grey pubescent markings, setal bases glossy black; scutellum overlain with sparse grey pubescence; pleuron orange to maroon, overlain with sparse silver-grey pubescence; wing markings dark banded infuscate; haltere knob white; coxae, femora and tibia orange-maroon; tarsi lighter orange, foreleg with basitarsus dark, white apically, rest of foretarsi white with slightly darker apex. Scutal chaetotaxy: *np*, 4–5; *sa*, 1; *pa*, 1; *dc*, 1; *sc*, 1. *Abdomen*. Dark, tergites 2–3 red-brown laterally, gold-bronze velutum on tergites 4–7; terminalia pale.

**Figure 24.**
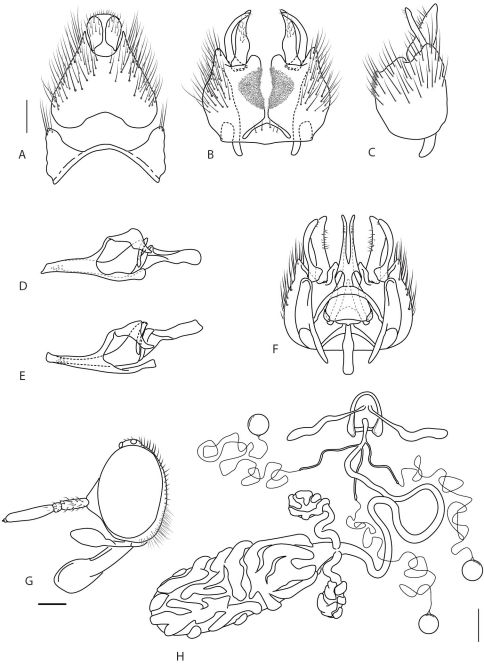
*Pipinnipons* spp.: **A** *Pipinnipons fascipennis* (Kröber), epandrium and tergite 8, dorsal **B** gonocoxites, ventral **C** same, lateral **D** aedeagus, lateral **E** *Pipinnipons kroeberi* Winterton, aedeagus, lateral **F** gonocoxites, epandrium removed and aedeagus *in situ*, dorsal **G** *Pipinnipons fascipennis*, male head, lateral **H** *Pipinnipons kroeberi*, female internal genitalia showing spermathecal sac complex. Scale lines = 0.2 mm.

**Figure 25.**
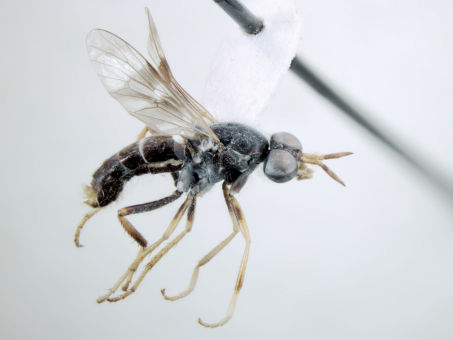
*Pipinnipons chauncyvallis* sp. n., male, lateral view [576261]. Body length = 7.0 mm.

#### Comments.

The gold-bronze abdominal velutum covering in both sexes and the dark orange pleuron and leg colouration make *Pipinnipons fascipennis* easily recognisable. This species is found in closed forest areas, including rainforest.

### Pipinnipons kampmeierae sp. n.

urn:lsid:zoobank.org:act:5F4F235B-49AE-431B-A79C-041BB73DE633

http://species-id.net/wiki/Pipinnipons_kampmeierae

Fig. 27

#### Type material.

**Holotype**male, AUSTRALIA: **Queensland:** Jimmy’s Scrub State Forest, nr. Goomeri, 22.xi.1985, M. De Baar (MEI165194) (QM). **Paratype**. AUSTRALIA: **Queensland:** male, Bribie Island, DPI Fisheries site, [-27.055°, 153.193°], 7.x.1997, S. L. Winterton, N. Power, D. White, heathland- *Acacia* regrowth, Malaise trap (MEI090764) (QM).

#### Diagnosis.

Wing mostly hyaline; pleuron black; coxae, femora and tibia orange; abdomen black, segments 2–3 orange with dark medial patch.

#### Description.

Body length= 6.0–7.0 mm. *Head*. Frons narrower than ocellar tubercle, profile rounded above antenna, surface texture smooth, glabrous; gena with pale setae; parafacial overlain with silver pubescence; palpus brown-black; occiput glabrous, glossy black; antennal base raised; antennal length approximately equal to head; scape orange-yellow, much shorter than flagellum, scape with sparse dark setae dorsally; flagellum orange-yellow, base of flagellum without setae. *Thorax*. Scutum glossy black, overlain with sparse yellow setae, grey pubescence laterally; scutellum overlain with sparse grey pubescence; pleuron black, overlain with sparse silver-grey pubescence; wing largely hyaline, faint band midway; haltere knob white; coxae, femora and tibia orange; tarsi white, fore-basitarsus dark, rest of basitarsi orange. Scutal chaetotaxy: *np*, 5; *sa*, 1–2; *pa*, 1; *dc*, 2; *sc*, 1. *Abdomen*. Segments 2–3 orange with black medial patch, remaining segments black; terminalia pale.

#### Comments.

*Pipinnipons kampmeierae* sp. n. is very similar to *Pipinnipons kroeberi*, but can be differentiated based on coxae and abdomen colouration. The wings are only weakly infuscate rather than strongly banded infuscate as in most other species of *Pipinnipons* and *Acupalpa*. The female is unknown for this species.

#### Etymology.

This species is named in honour of Gail Kampmeier, in recognition of her excellent work on Therevidae bioinformatics.

### Pipinnipons kroeberi

Winterton

urn:lsid:zoobank.org:act:97517024-09B0-41E4-8E27-DA0D8A8155CF

Genbank Accession: AF150980

http://species-id.net/wiki/Pipinnipons_kroeberi

Figs 24E–F 28

Pipinnipons kroeberi  Winterton, 2001: 205.

#### Type material.

**Holotype**male,AUSTRALIA: **New South Wales:** Warrumbungle N.P., Buckleys Creek, 1.7 km N Camp Blackman, 23.xii.1992 M. E. Irwin (MEI027580) (ANIC). **Paratypes.** AUSTRALIA: **New South Wales:** female, Warrumbungle N.P., Browns Creek, 2.5 km N Woolshed, 13.i.1994, M. E. Irwin (MEI039303) (ANIC). **Queensland:** male, female, Lake Broadwater, 25 km SW Dalby, on *Leptospermum flavescens* blossom, 18.x.1985, D. K. Yeates (MEI090894, 090895) (GDCB/AMS).

#### Diagnosis.

Wing hyaline; pleuron black; femora and tibia orange to yellow; abdomen orange, segments 1–3 sometimes black medially.

#### Redescription.

Body length= 8.0–9.5 mm. *Head*. Frons wider than ocellar tubercle (equal in male), profile rounded or transversely concave above antennae (female), pubescence as silver patches along eye margin, frons otherwise glabrous, surface texture as irregular longitudinal striations (female) or smooth (male); gena with dark setae; parafacial overlain with silver pubescence; palpus yellow-orange; occiput glabrous, glossy black; antennal base raised; antenna longer than head; scape yellow, length much shorter than flagellum, scape with sparse pale setae; flagellum yellow, base of flagellum with short, dark setae. *Thorax*. Scutum uniform grey-black; scutellum overlain with sparse, grey pubescence; pleuron black, overlain with sparse, silver-grey pubescence; wing hyaline, orange suffusion along costal margin; haltere knob orange-yellow; coxae orange-yellow (hind coxa dark); femora and tibia orange or yellow, fore tibia apex dark; tarsi yellow, fore-basitarsus dark basally, rest of foreleg tarsomeres white. Scutal chaetotaxy: *np*, 4; *sa*, 1; *pa*, 1; *dc*, 3; *sc*, 1. *Abdomen*. Orange, segments 1–2 black medially; terminalia pale.

#### Comments.

*Pipinnipons kroeberi* is similar to *Pipinnipons kampmeierae* sp. n. and can be differentiated based on the body colouration. This speciesis found in southeastern Queensland and northeastern New South Wales.

### Pipinnipons sphecoda sp. n.

urn:lsid:zoobank.org:act:329B77AF-DF06-4FB5-9E0C-0EEB79F1638F

http://species-id.net/wiki/Pipinnipons_sphecoda

Fig. 29

#### Type material.

**Holotype**female,AUSTRALIA: **Tasmania:** Claytons [-43.383°, 146.133°], Jan.1991, E. D. Edwards, E. S. Nielsen (MEI027583) (ANIC). **Paratypes**. AUSTRALIA: **Tasmania: **female, Lake St. Clair, [-42.067°, 146.167°], 25.i.1949, E. F. Riek (MEI027585) (ANIC); female, 10 km ENE Nunamara [-41.367°, 147.4°], 12.i.– 7.ii.1983, malaise trap, I. D. Naumann, J. C. Cardale (MEI027586) (ANIC).

#### Diagnosis.

Wing with yellow and black irregular banding; pleuron black; femora and tibia yellow, sometimes with dark suffusion midway along femur; abdomen black, bright yellow-orange markings on tergites 1–4 and 7–8.

#### Description.

Body length= 10.0–14.0 mm. *Head*. Frons wider than ocellar tubercle, profile slightly transversely concave above antennae, pubescence absent, frontal vestiture as minute setae, surface texture as irregular longitudinal striations and transverse striations; gena with dark setae; parafacial overlain with silver pubescence; palpus yellow-orange; occiput glabrous, glossy black; antennal base raised; antennal length approximately equal to head; scape yellow, shorter than flagellum, scape with sparse black setae; flagellum yellow, base of flagellum with short dark setae. *Thorax*. Scutum glossy black, overlain with sparse yellow setae, sparse grey pubescence laterally; scutellum overlain with dense, matt-black pubescence; pleuron black, overlain with sparse silver-grey pubescence; wing with dark band midway, pale yellow suffusion basally and in discal area; haltere knob white to yellow; coxae black, overlain with silver pubescence; femora yellow-orange, sometimes with dark patch midway; tibia yellow-orange; tarsi yellow, fore leg with basitarsus and second tarsomere white, rest of tarsomeres dark. Scutal chaetotaxy: *np*, 4–5; *sa*, 1; *pa*, 2; *dc*, 1; *sc*, 1. *Abdomen*. Black, bright yellow-orange markings on tergites 1–4 and 7–8; terminalia pale.

#### Comments.

*Pipinnipons sphecoda* sp. n. is a relatively large, apparently wasp-mimicking species known only from female specimens collected from various sites in Tasmania. The dramatic colouration of species makes it quite unlike any other stiletto fly species.

#### Etymology.

The species epithet is derived from the Gr. *sphekodos*, wasp-like.

**Figure 26.**
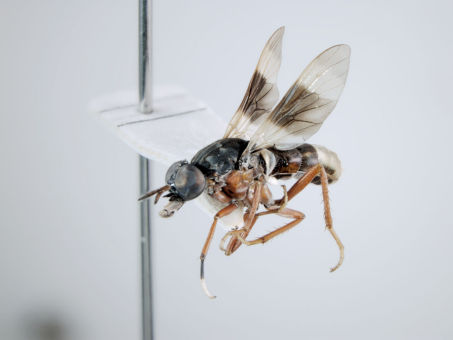
*Pipinnipons fascipennis* (Kröber), male, anterolateral view [576262]. Body length = 7.0 mm.

**Figure 27.**
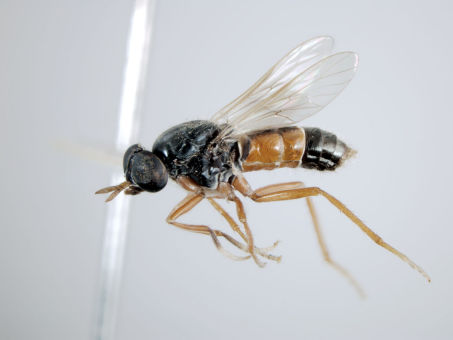
*Pipinnipons kampmeierae* sp. n., male, lateral view [576263]. Body length = 6.0 mm.

**Figure 28.**
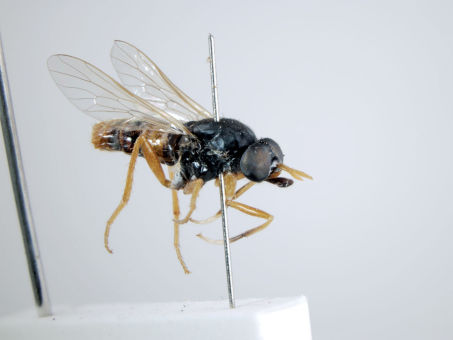
*Pipinnipons kroeberi* Winterton, male, anterolateral view [576264]. Body length = 8.0 mm.

**Figure 29.**
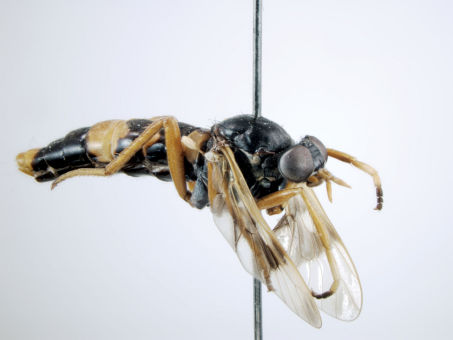
*Pipinnipons sphecoda* sp. n., female, anterolateral view [576265]. Body length = 10.0 mm.

## Supplementary Material

XML Treatment for Acupalpa

XML Treatment for Acupalpa albimanis

XML Treatment for Acupalpa albitarsa

XML Treatment for Acupalpa boharti

XML Treatment for Acupalpa divisa

XML Treatment for Acupalpa dolichorhyncha

XML Treatment for Acupalpa glossa

XML Treatment for Acupalpa imitans

XML Treatment for Acupalpa irwini

XML Treatment for Acupalpa melanophaeos

XML Treatment for Acupalpa miaboolya

XML Treatment for Acupalpa minuta

XML Treatment for Acupalpa minutoides

XML Treatment for Acupalpa notomelas

XML Treatment for Acupalpa novayamarna

XML Treatment for Acupalpa rostrata

XML Treatment for Acupalpa semirufa

XML Treatment for Acupalpa westralica

XML Treatment for Acupalpa yalgoo

XML Treatment for Acupalpa yanchep

XML Treatment for Pipinnipons

XML Treatment for Pipinnipons chauncyvallis

XML Treatment for Pipinnipons fascipennis

XML Treatment for Pipinnipons kampmeierae

XML Treatment for Pipinnipons kroeberi

XML Treatment for Pipinnipons sphecoda
